# Comparing the Functional Independence Measure and the interRAI/MDS for use in the functional assessment of older adults: a review of the literature

**DOI:** 10.1186/1471-2318-9-52

**Published:** 2009-11-29

**Authors:** Christine Glenny, Paul Stolee

**Affiliations:** 1 Department of Health Studies and Gerontology, University of Waterloo (200 University Avenue East), Waterloo (N2L 3G1), Canada

## Abstract

**Background:**

The rehabilitation of older persons is often complicated by increased frailty and medical complexity - these in turn present challenges for the development of health information systems. Objective investigation and comparison of the effectiveness of geriatric rehabilitation services requires information systems that are comprehensive, reliable, valid, and sensitive to clinically relevant changes in older persons. The Functional Independence Measure is widely used in rehabilitation settings - in Canada this is used as the central component of the National Rehabilitation Reporting System of the Canadian Institute of Health Information. An alternative system has been developed by the interRAI consortium. We conducted a literature review to compare the development and measurement properties of these two systems.

**Methods:**

English language literature published between 1983 (initial development of the FIM) and 2008 was searched using Medline and CINAHL databases, and the reference lists of retrieved articles. Relevant articles were summarized and charted using the criteria proposed by Streiner. Additionally, attention was paid to the ability of the two systems to address issues particularly relevant to older rehabilitation clients, such as medical complexity, comorbidity, and responsiveness to small but clinically meaningful improvements.

**Results:**

In total, 66 articles were found that met the inclusion criteria. The majority of FIM articles studied inpatient rehabilitation settings; while the majority of interRAI/MDS articles focused on nursing home settings. There is evidence supporting the reliability of both instruments. There were few articles that investigated the construct validity of the interRAI/MDS.

**Conclusion:**

**A**dditional psychometric research is needed on both the FIM and MDS, especially with regard to their use in different settings and with different client groups.

## Background

Measurement and reporting health outcomes have become an essential component guiding the development and evolution of health care systems. As the focus of health care changes to adapt to the aging population, aggregate data from health assessment systems can be used to inform policy decisions regarding service use and best practices [1]. One health care setting that serves a primarily older clientele is post acute rehabilitation [2]. There is a substantial need for accurate assessment in this population as it can have significant implications for older patients' care planning and future quality of life [3]. Despite some encouraging research in this area [4-6], there is limited data that focus on measuring rehabilitation outcomes in older adults [7]. One major challenge is that the performance of currently available assessment systems is not well understood in this population.

Development of valid and reliable outcome measures for use with older adults is complicated by frailty, comorbidity, and heterogeneity in this population. Geriatric patients are different from their younger counterparts as they tend to have lower functional status on admission and higher clinical complexity due to multicausal disability and intercurrent medical conditions [4,8,9]. Older adults are an extremely diverse population and represent a wide range of physical and cognitive abilities [2]. Individualized measures, such as Goal Attainment Scaling, have been suggested as a possible approach to address this heterogeneity [10], however such measures present challenges for the development of a consistent database of client information. Wells and colleagues [8] recommend that standardized tools should be used for diagnosis, assessment, and outcome measurement in geriatric rehabilitation. Instruments that are designed for younger, healthier, and more homogenous groups are unlikely to have the same psychometric properties with older adults [2] and additional research is required specifically related to the performance of assessment tools and outcome measures in older populations of rehabilitation patients.

Both the Functional Independence Measure (FIM) [11] and the interRAI/Minimum Data Set (MDS) [12,13] are instruments designed to measure functional ability, and both have been used widely with older persons and are mandated in multiple health care settings. Specific components of these instruments collect parallel information and items on both the FIM and the MDS can be used to predict total scores on the other tool [14,15].

In spite of their similarities, the range of content coverage, item definitions, scoring, and psychometrics are not identical for both tools, which prevents direct translation of scores from one instrument to another [16,17]. Comparative information on their psychometric properties would be helpful in assessing the relative merits and potential applications of the two instruments. The purpose of this investigation was to examine previously published research on the measurement properties of these tools for use in populations of older adults.

## Instruments

### Functional Independence Measure (FIM)

The FIM was developed in 1983 by a task force created by the American Congress of Rehabilitation Medicine and the American Academy of Physical Medicine and Rehabilitation headed by Carl Granger and Byron Hamilton [11]. To generate items, this group conducted a literature review of 36 existing functional performance measures [8]. The final instrument was based on the Barthel Index [18,19], which has been in use since the 1950s [20]. The FIM was designed to measure physical and cognitive disability and focuses on burden of care [11]. The main objective in its development was to create a generic measure that could be administered by clinicians and non-clinicians to assess patients in all age groups with a wide variety of diagnoses [11]. The FIM contains a total of 18 items. Thirteen of these items constitute the motor subscale and the remaining five items form the cognitive subscale [21]. The motor subscale collects information involving self care, sphincter control, transfer, and locomotion, and the cognitive subscale focuses on communication and social cognition. All items are scored using a seven-point ordinal scale that is based on the amount of assistance that is required for the patient to perform each activity [21]. Higher scores on the FIM denote patients that have a higher level of independence and require a small amount of assistance [21]. The sum of all 18 items gives the patient's total score, which ranges from 18-126 [21]. The FIM is the major source of functional status data in the National Rehabilitation Reporting System (NRS) of the Canadian Institute for Health Information [22].

### interRAI/Minimum Data Set (MDS)

interRAI is an international research consortium that develops comprehensive assessment tools that are principally intended for older adult populations [13,23]. These Resident Assessment Instruments (RAIs) are used internationally in a wide variety of health care settings for a large number of applications including care planning, outcome measurement, and quality indicators [24]. Currently, there are 12 RAI tools designed for use in rehabilitation, long term care, home care, and other settings across the health care continuum [23]. The instruments consist of over 300 items encompassing a large array of patient characteristics including functional status, admission history, medical conditions and other information [24]. Initially these items were generated by reviewing previous literature on over 60 assessment instruments [25]. The final sets of items were selected based on extensive clinical deliberations and an iterative review process mainly focused on interrater reliability and clinical relevance [25]. All of the tools contain a proportion of common items that are intended to facilitate communication across multiple health care settings [26,13]. Each individual tool also includes specialized items exclusive to that setting [26]. The instrument specifically designed for use in rehabilitation is the interRAI Post Acute Care [27,23].

Physical functioning is measured by a range of activities of daily living (ADL) items that can be summed to form several ordinal ADL scales [28]. These items were designed to measure activities across a wide range of functional independence levels to enable the detection of functional changes in individuals with both high and low levels of functioning [28]. Each item is scored on the basis of the amount of assistance required for performance, with higher scores indicating greater dependence [28]. The scales were developed based on exploratory factor analyses and hypothesis testing to arrange the ADL items hierarchically in relation to loss of functioning. Currently there has been no consensus on a single standard ADL subscale for the interRAI instruments [28-32].

Cognitive functioning can be estimated using the interRAI instruments in two ways - the 5-item Cognitive Performance Scale [CPS; [33]] or the 11-item MDS Cognition Scale (MDS-COGS) [34]. Both scales are ordinal with the CPS ranging from 0 (intact) to 6 (very severe impairment) and the MDS-COGS ranging from 0 (cognitively intact) to 10 (very severe impairment). These scales were both developed based on their correlation with and ability to predict scores of existing cognition scales, including the Mini-Mental State Exam [35], Test for Severe Impairment [36] and the Global Deterioration Scale [34,33,37].

## Methods

### Criteria for considering studies in this review

All relevant English language articles that were published between January 1983 (the initial year of development for the FIM) and June 2008 were included in this review. The following inclusion and exclusion criteria were established to determine article relevance:

#### Inclusion criteria

1) The study population included older adults (55+)

2) The main focus of the article was on some aspect related to the development and/or measurement properties of the FIM and/or MDS instruments

#### Exclusion criteria

1) The article focused on child, adolescent, and/or young adult populations

2) The article did not contain original data, statistical analyses, and/or results

3) The article was a review of previously published work

4) The article solely focused on patients with spinal cord injuries and/or traumatic brain injuries

5) The article was focused on reports of experimental versions of the FIM and/or MDS or reported assessments of the properties of additional items or short forms that are not currently used in clinical practice

6) The instruments were used in the study as an intervention (e.g. instrument used to test the effects of a comprehensive assessment on patient outcomes)

7) The article did not relate to MDS items or subscales that are comparable to FIM items

### Search methods for identification of studies

Electronic searches

Published material was identified using the MEDLINE and CINAHL databases using the following search strategy:

#### MEDLINE database

1)Functional Independence Measure [TIAB] OR FIM [TIAB] Limits: Published in 1983 to 2008

2) Minimum Data Set [TIAB] OR MDS [TIAB] OR interRAI [TIAB] OR Resident Assessment Instrument [TIAB] Limits: Published in 1983 to 2008

3) Reproducibility of results [MeSH] OR reliability [TIAB] OR interrater [TIAB] OR intrarater [TIAB] OR test retest [TIAB] OR internal consistency [TIAB] OR validity [TIAB] OR criterion [TIAB] OR construct [TIAB] OR content [TIAB] OR responsiveness [TIAB] OR clinically relevant change [TIAB] OR clinically important change [TIAB] OR development [TIAB] OR psychometric [TIAB] OR performance [TIAB] OR validation [TIAB] OR dimentionality [TIAB] OR structure [TIAB] Limits: Published in 1983 to 2008

4) Delirium, dementia, amnestic, cognitive disorders [MeSH] OR activities of daily living [MeSH] OR functional assessment [TIAB] OR cognitive [TIAB] OR cognitively [TIAB] OR cognitive performance scale [TIAB] OR function [TIAB] OR physical [TIAB] OR activities of daily living [TIAB] OR ADL [TIAB] OR motor function [TIAB] Limits: Published in 1983 to 2008

5) 1 AND 3 AND 4

6) 2 AND 3 AND 4

#### CINAHL database

1) Functional Independence Measure [TIAB] OR FIM [TIAB] Limits: Published in 1983 to 2008

2) Minimum Data Set [TIAB] OR MDS [TIAB]OR interRAI [TIAB] OR Resident Assessment Instrument [TIAB] Limits: Published in 1983 to 2008

3) Reliability and validity [MH+] OR reliability [TIAB] OR interrater [TIAB] OR intrarater [TIAB] OR test retest [TIAB] OR internal consistency [TIAB] OR validity [TIAB] OR criterion [TIAB] OR construct [TIAB] OR content [TIAB] OR responsiveness [TIAB] OR clinically relevant change [TIAB] OR clinically important change [TIAB] OR development [TIAB] OR psychometric [TIAB] OR performance [TIAB] OR validation [TIAB] OR dimentionality [TIAB] OR structure [TIAB] Limits: Published in 1983 to 2008

4) Delirium, dementia, amnestic, cognitive disorders [MH+] OR activities of daily living [MH+] OR functional assessment [TIAB] OR cognitive [TIAB] OR cognitively [TIAB] OR cognitive performance scale [TIAB] OR function [TIAB] OR physical [TIAB] OR activities of daily living [TIAB] OR ADL [TIAB] OR motor function [TIAB] Limits: Published in 1983 to 2008

5) S1 AND S3 AND S4

6) S2 AND S3 AND S4

#### Manual searches

The reference lists of the retrieved articles were examined for additional relevant papers.

### Data collection and analysis

Guided by the inclusion and exclusion criteria, the first author eliminated irrelevant articles based on the title of the publication and the content of its abstract. All potentially relevant articles were retrieved and reviewed. Any article that was retrieved but was later found to be potentially irrelevant was reviewed by the second author. When the relevance was questionable, the two authors discussed the paper to arrive at a final conclusion. For each of the selected articles, information was gathered and charted according to the reliability and validity criteria proposed by Streiner [38].

## Results

**Figure 1:**
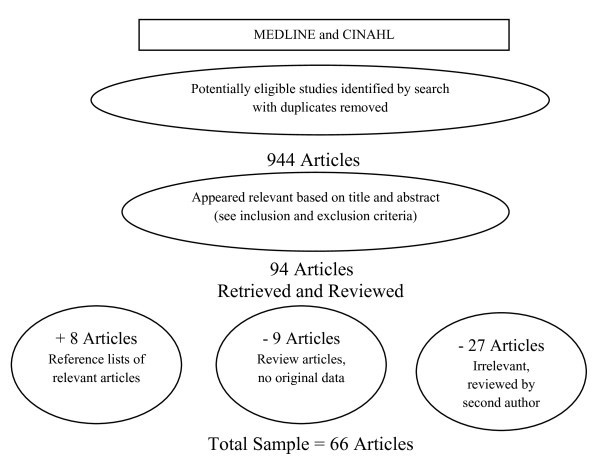
**Results of search strategy**.

The initial keyword search identified 944 articles, of which 850 were excluded based on review of the title and abstract. Ten additional articles were identified by hand-searching the reference lists of articles obtained in the initial search. Of the 94 articles retrieved for further review, 24 were excluded based on relevance and 12 were excluded as they were reviews of previously published works (Figure 1).

Forty articles focused on the FIM, 26 focused on the MDS, and 1 article investigated both instruments. Tables 1, 2 S3, S4, S5 and S6 (Tables S3, S4, S5, and S6 can be found in Additional file 1) summarize the total sample of articles that met the criteria for this review [11,16,25-29,31,32,34,39-93].

**Table 1:** Summary of validity and reliability studies for the FIM and MDS

	Reliability				Validity				
	
	Internal Consistency	Intrarater	Interrater	Total	Criterion	Construct	Content	Face	Total
**FIM**	6	2	5	**13**	14	26	0	1	**41**

**MDS/interRAI**	5	1	12	**18**	12	7	0	1	**20**

* Some articles discuss multiple types of reliability and validity; therefore, totals do not correspond with the total number of articles in the sample

**Table 2:** Definitions of key terms

**Reliability**: indicator of the tool's consistency	**Validity**: determines whether the tool measures what it was designed to measure
**Internal consistency**: measures the average correlation between all items on a tool **Intrarater reliability**: an indicator of the tests' stability overtime when it is administered by the same rater **Interrater reliability**: indicates the consistency of a tool when it is administered by different raters	**Construct validity**: investigates whether the tool correlates with a theorized construct **Criterion validity**: can be divided into two categories; concurrent and predictive. Concurrent criterion validity measures the correlation of the tool with other tools that measure the same concepts, preferably a "gold standard" when it exists. Predictive criterion validity examines whether the tool can predict future outcomes. **Content validity**: assesses whether the tool targets all of the relevant topics related to the concept being measured and that there are no irrelevant items **Face validity**: an assessment of whether the tool appears to measure the intended concept

* Some articles discuss multiple types of reliability and validity; therefore, totals do not correspond with the total number of articles in the sample

A nearly equal number of FIM articles investigated internal consistency and interrater reliability, while most MDS articles focused on interrater reliability. For both instruments, few articles investigated intrarater reliability. Four of the FIM articles focused on inpatient rehabilitation populations and five studied community residents mostly receiving home care. A large majority of MDS articles focused on nursing home residents and no articles were found that solely focused on inpatient rehabilitation. Clinicians were commonly used as raters for both instruments; three FIM and two MDS articles used researchers to assess the participants.

Internal consistency was high for the FIM total score (α = 0.88-0.97), domains (motor α = 0.86-0.98, cognitive α = 0.68-0.95), and subscales (α = 0.68-0.96); and the FIM was found to have greater consistency than other tools commonly used in inpatient rehabilitation [44]. Dallmeijer and colleagues [39] concluded that the FIM motor has slightly higher internal consistency than the FIM cognitive; however, this result was not replicated in other studies [45,51]. Multiple studies found slight variations in internal consistency between impairment types [39,51,41]. Two articles investigated the intrarater reliability of the FIM. In both articles, the participants were assessed by researchers, and both concluded that the FIM total and domains have very high reliability [FIM total r = 0.94-0.98, motor r = 0.90-0.97, cognitive r = 0.80-0.99; [48,40]]. Five additional articles also concluded that the FIM was reliable when they focused on interrater reliability (FIM total ICC = 0.80-0.99, FIM motor ICC = 0.91-0.99, FIM cognitive ICC = 0.91-0.99). The interrater reliability was highest when both raters were present at the same interview, raters participated in FIM training prior to conducting their first assessment, raters met Uniform Data System for Medical Rehabilitation (UDS_MR_) criteria, and the testing period was short [39,40,42,43,47,48].

During the development of MDS instruments, unreliable items were progressively eliminated resulting in increasing reliability estimates over time [25,81]. Five articles investigated the internal consistency of functional status related outcome measures in the MDS. In all five studies, the researchers concluded that the scales(s) investigated was(were) internally consistent. However, because many of the characteristics - including subjects, setting, and raters - are different between the studies, and reliability is dependent on such variations [94], it is not currently possible to develop generalizations across these articles about patterns in consistency. Zimmerman and colleagues [87] were the only group to investigate the intrarater reliability of an MDS subscale. They found that the relative amount of within and between rater error changed for the MDS-COGs depending on which cut-point was used. High interrater reliability has been repeatedly shown for MDS items in nursing home settings (Individual items r = 0.75-0.99, κ = 0.56-0.84, wκ = 0.33-1.0). Many of these studies investigated the reliability of MDS items in isolation and did not assess the reliability of summative scales within the instrument. Across all types of reliability, when summative scales were investigated, there was a lack of consistency in the MDS items used to form cognitive and ADL subscales.

Sixty-one of the articles in the sample investigated the validity of the instruments. The FIM and the MDS were independently discussed in 41 and 20 articles respectively. This difference was mainly due to the notably larger proportion of FIM articles that focused on construct validity. Eight articles investigated the responsiveness of the FIM and only three articles investigated the responsiveness of the MDS - there was considerably more evidence supporting the responsiveness of the FIM than the MDS. The majority of FIM articles focused on inpatient rehabilitation and the remaining studied populations in a variety of health care settings including home care, neurorehabilitation, nursing homes, and acute care. Almost three quarters of the MDS articles investigated the validity of the tool in nursing home residents; no articles exclusively focused on patients in rehabilitation settings.

For both instruments, face validity was investigated during development and early implementation [11,25]. To examine the face validity of the FIM, a wide variety of raters (including: occupational therapists, physiotherapists, nurses, doctors, speech pathologists, recreation therapists, social workers, and researchers) assessed patients from an inpatient rehabilitation facility [11]. Following their assessment, each rater was surveyed regarding the necessity of each FIM item and the adequacy of the total scale [11]. This resulted in the revision of multiple existing items, the addition of two new items, and the increase of response options from four to seven [11]. Ten FIM articles assessed concurrent criterion validity. Three of these focused on alternative methods of FIM administration and found that caregivers of home care patients can accurately report FIM items, and patient or nurse interviews are useful assessment alternatives to direct patient observation in a neurorehabilitation setting [54,58,59]. Seven articles focused on the correlation of the FIM with other functional assessment instruments. They found that the FIM correlates with various instruments used in home care, acute care, and inpatient rehabilitation including the BI and the Functional Autonomy Measurement System [44,47,48,52,55,60,95]. Four articles investigated predictive criterion validity of the FIM and found that in a home care setting the FIM can predict burden of care but not life satisfaction, and in inpatient rehabilitation settings the FIM can consistently predict discharge location, length of stay, and discharge function [53,64,68,73].

Of the twenty-six articles that assessed the construct validity of the FIM, seven used factor analysis to investigate the instruments dimensionality. Three of the seven articles concluded that the FIM has a bidimensional structure defined by the motor and cognitive domains [39,55,51], and the remaining four articles concluded that the FIM has a multidimensional structure defined by three to five factors which were often related to either the subscales within the instrument or to anatomical regions (e.g., lower or upper body) [45,46,50,62]. All of the articles consistently found the cognitive domain to have a unidimensional structure and any additional factors were contained in the motor domain [45,46,50,62]. Eight articles investigated the construct validity of the FIM using Rasch analysis. These had mostly consistent findings: eating and stair climbing were seen to be the easiest and most difficult FIM motor items respectively; expression and problem solving are the easiest and most difficult FIM cognitive items; bowel, bladder, eating, and stair climbing are common "misfit" items on the FIM motor; the distribution of FIM scores has a sigmoidal structure and the number of response options should be reduced [21,39,49,66,67,69-71]. The three articles that assessed the dimensionality of the FIM using Rasch support bidimensional constructs defined by the motor and cognitive domains [21,49,66]. Six articles used Rasch analysis to investigate differential item functioning [22,39,66,67,69,70]. These articles consistently found evidence of DIF between impairment groups; however, they disagreed on its clinical relevance. Eight articles investigated the responsiveness of the FIM; most estimated clinically relevant change using effect size and standardized response mean statistics. All of these articles focused on patients in neurorehabilitation or inpatient rehabilitation settings and consistently found that the FIM total, FIM motor, and FIM motor subscales are responsive and the FIM cognitive and FIM cognitive subscales are not responsive in this population [41,44,52,57,60,74,76,77]. The FIM was also found to be as responsive as other functional assessment instruments used in inpatient rehabilitation including the BI.

Similar to the FIM, one article formally assessed the face validity of the MDS [25]. In a nursing home setting, following resident assessment with the MDS, trained nurses were asked to comment on the relevance of each MDS item and their response options [25]. The nurses felt that the multi-category items were crucial for care planning and a one-point difference on each item represented a clinically relevant change [25]. The strong majority of MDS articles focused on concurrent criterion validity. These articles repeatedly found scores on the CPS, MDS-COGS, and a variety of ADL subscales to correlate with other instruments commonly used in home care and nursing homes including the MMSE, GDS, Lawton Index [96], and the BI [32,83].

Of the four articles that focused on construct validity, one investigated the structure of the MDS using a confirmatory factor analysis [79]. They found that the factor structure was different in groups of nursing home clients depending on their level of cognitive impairment [79]. The remaining three articles examined the responsiveness of the MDS and each used different criteria for defining clinically relevant change in populations of nursing home residents. Carpenter and colleagues [88] defined a one-point change as clinically meaningful (based on Morris and collegues, 1990) and found that the ADL-Long Form was responsive over three months and six months. Morris and colleagues [28] collected data on longitudinal change rates in nursing home residents and based on average expected decline defined clinically meaningful change as 4% of one standard deviation over three months and 13% of one standard deviation over six months, and also found three ADL scales contained within the MDS [the ADL-long form, ADL-short form, and ADL-hierarchy scale [28] to be responsive. Lastly, Snowden and colleagues [92] used effect size to estimate the responsiveness of the CPS and a 6-item summative ADL subscale in nursing home residents enrolled in the Alzheimer's Disease Patient Registry [ADPR; [33]]. They concluded that the CPS (ES = 0.60) was slightly more responsive and the ADL subscale [ES = 0.024] was significantly less responsive than the cognition (MMSE ES = 0.39) and ADL (Dementia rating Scale; DRS ES = 0.77) outcome measures currently used by the ADPR [92].

Only one study directly compared the FIM and the MDS 2.0 in the same article [16]. Using Rasch analysis, these researchers investigated whether setting specific functional assessment instruments (FIM, OASIS, MDS 2.0 and PF-10 (ADL component of the Short Form-36)) used in post acute care contain differences that prevent their use across different health care settings [16]. Data were mostly obtained from retrospective chart review and samples were compared where each participant was assessed for one of the outcome measures of interest. They found that many FIM and MDS items cluster around the centre of the functional difficulty range, the range of content coverage was wider for the MDS than the FIM, and the MDS measures functional ability most precisely at the low end of the dimension whereas the FIM is more precise in the low to moderate dimension [16]. They concluded that both instruments were well suited for their specific application but neither instrument is well equipped across all settings [16].

## Discussion

The purpose of this review was to accumulate and synthesize past research focusing on the reliability and validity of the FIM and the MDS for use with older adults. To our knowledge, there have been no publications to date that have systematically reviewed and compared evidence of the psychometric properties of both tools. It is important for functional status outcome measures to be validated for use with older adults because this group of individuals represents a substantial proportion of the population being assessed with these instruments. Also, it is unlikely that the measurement properties of assessment tools will be consistent between the older and younger populations [2,38,94].

For both the FIM and the MDS, the majority of articles used samples from the same type of health care setting. Over half of the FIM studies were conducted in inpatient rehabilitation settings and almost two-thirds of the MDS articles were conducted with nursing home residents. Also, as MDS instruments are composed of similar items, psychometric data for a single MDS instrument, usually the MDS 2.0, were often extrapolated to other MDS instruments. This may not be appropriate as reliability and validity estimates are dependent on variation in the sample on which the instrument was tested [94] and the individual MDS instruments are designed to be used on samples with different characteristics. This implies that while the MDS instruments have excellent reliability estimates in a sample of nursing home residents, these results might not be obtained in a different sample with dissimilar characteristics. In a recent study, however, Hirdes and colleagues [26] showed that the reliability of individual MDS items was consistent across multiple settings. This study provides important evidence supporting the reliability of the MDS in applications across the health care continuum. Nonetheless, as both the FIM and the MDS are designed to be generic instruments, future research with both instruments is needed in a wider range of health care settings to determine if their psychometric properties are equivalent across different settings and client groups.

Many of the MDS articles focused on how consistently individual items were scored and not on the reliability of embedded outcome measures such as the CPS and the various ADL scales. This may be a result of intentions to preserve the ability to use various combinations of individual items over time and across different settings, while retaining evidence of their reliability. When the properties of physical and cognitive outcome measures in the MDS were assessed, the investigators tended to use different numbers and combinations of items. These inconsistencies are problematic because scales that contain different items may have different measurement properties [94] therefore making it difficult to accumulate and compare the results from multiple studies. More research is needed to develop or select a consistent ADL subscale for the MDS.

Consistent with the findings of other reviews [97,98], we found substantial evidence of the reliability and validity of the FIM and of the reliability of the MDS. Contradictory evidence was found regarding the internal consistency of the FIM in different impairment groups. In an inpatient rehabilitation setting, Dodds and colleagues [41] found that the internal consistency of FIM items varied by impairment group, especially for the locomotion subscale. This may suggest that all FIM items are not relevant for all impairment types, or that the instrument is not functioning consistently for different types of patients [38]. Conversely, Stineman and colleagues [51] investigated this relationship in a sample of community residents and concluded that internal consistency was excellent and no items should be removed for any of the 20 UDS_MR _impairment types. The inconsistency between these two articles may be due to different distributions and severities of impairment types in inpatient rehabilitation and community settings. This may suggest that all FIM items are relevant in higher functioning groups (community residents) but not in lower functioning groups (patients in inpatient rehabilitation). Multiple studies identified DIF by impairment group, which also supports this hypothesis [21,39,66,67,69,70].

For both the FIM and the MDS, few articles were located that investigated intrarater reliability. Traditionally, it is more practical and economical to assess interrater reliability as it includes more sources of error: the raters are different and the participant being assessed may have changed over the testing period [38]. As a result, intrarater reliability is necessary but not sufficient for interrater reliability. However, intrarater reliability can be used to further investigate the source of low interrater reliability. For example, if an instrument has low interrater reliability and high intrarater reliability it may mean that the raters have been trained inadequately, resulting in inconsistent evaluations [38]. Daving and colleagues [40] used clinicians to investigate the reliability of the FIM in community residents. They found that the reliability ranged from poor to excellent where the least reliable assessments were completed at different times by different raters. As the interrater reliability of the FIM was generally high in other settings, an intrarater reliability study should be conducted to determine if clinicians assessing community residents are the source of this inconsistency. For both of the articles that investigated the intrarater reliability of the FIM, the raters were not clinicians. As researchers have different background knowledge and may receive different, more intense training programs prior to conducting assessments, this may have artificially inflated the results leading to the high and more narrow range of estimates. Using researchers instead of clinician raters also limited their investigation of the source of error in the natural environment.

Streiner and Norman [94] assert that validity evidence from a series of converging experiments is superior to the results of one study. This is due to the inability of a single study to investigate definitively all aspects of an instrument's hypothetical construct; conclusions regarding the validity of an instrument may vary with the sample, setting and many other factors [38,94]. Therefore, the validity of an instrument is established by the accumulation of evidence across multiple studies. In this sample, there were twice as many studies investigating the validity of the FIM as the MDS. Both the FIM and the MDS have been repeatedly shown to correlate with commonly used assessment instruments in this area. However, because the outcome measures contained in both instruments were developed using these previously existing assessment tools [18,19,33,34] and there is no 'gold standard' instrument for measuring functional status in older adults, these investigations are not sufficient to establish the validity of either instrument. Relative to the FIM articles, the MDS articles were especially lacking in studies that focus on construct validity. There is a need for future research to investigate the construct validity of functionally related outcome measures contained in the MDS, including assessment of dimensionality, floor and ceiling effects, differential item functioning and responsiveness. Additional research is also needed on the construct validity of the FIM to investigate inconsistent findings regarding dimensionality and differential item functioning.

Determining the responsiveness of tools used to measure functional status in older adults is important because small scale changes may represent very large, clinically relevant, changes in quality of life. For example, a small change on a tool's scale can mean the difference between discharge to a long-term care facility or to home care. A number of methods have been proposed for the analysis of responsiveness [99,100]; however there is currently no consensus on a "gold standard" measure of responsiveness [99]. As a result, it is suggested that multiple measures of responsiveness be used in a single study to allow for the interpretation of patterns across different recommended statistics [101]. The methods used to measure the responsiveness of the FIM and the MDS differed widely across studies and very few studies applied more than one responsiveness statistic to the same sample. More research is needed to determine the responsiveness of the FIM and MDS.

Several limitations of this research are recognized. Although a detailed search strategy was developed to locate articles that fit the criteria for this review, it is possible that studies that did not principally focus on the psychometric properties of the MDS or the FIM could contain additional information on the reliability and validity of the tools. Also, all studies meeting the inclusion/exclusion criteria were included in the review regardless of their methodological merit. As we were aware of no prior attempt to collect and synthesize this information our aim was to be as comprehensive and inclusive as possible. Lastly, this review did not address the accreditation or training requirements, labour or time requirements for completion, software costs, and other administrative expenses, associated with either instrument. These would clearly be relevant considerations for organizations considering adoption of one of these instruments.

## Conclusion

This review assembled and compared available evidence of the reliability and validity of two major systems for the functional assessment of older adults. Overall, we found that there is evidence for the reliability of both instruments; however, the majority of FIM studies were carried out in inpatient rehabilitation settings and most of the MDS articles were conducted with nursing home residents. Before clinicians can confidently use the instruments outside of these settings, additional psychometric research is needed on both the FIM and MDS, especially with regard to their use in different settings and in different client groups. We also found that there is considerably more literature examining the validity of the FIM than is available for the MDS instruments. This supports the continued used of the FIM as a component of the NRS. Nonetheless, it is also important to consider that this analysis only included the ADL and cognition items from the MDS which contains a more comprehensive set of items that may enhance its utility. The compatibility of the interRAI instruments across multiple health care setting should also be considered before determining which tool is the most appropriate outcome measure for this population. We suggest that, in particular, more research is needed to investigate the construct validity of the outcome measures derived from the MDS instruments. Lastly, a direct "head to head comparison" of both tools in the same population would yield valuable information, especially in terms of the assessment of their responsiveness to change. Such a study could also allow for analysis (using Rasch methods, for example) that would facilitate direct statistical comparison of results obtained using the two instruments. While such analyses could theoretically lead to the development of a hybrid instrument, it is unlikely that such an instrument would gain broad acceptance given the extensive investments already made into the two systems. It is more likely that the results would facilitate better understanding of the results of each instrument by users of the other system.

## Competing interests

The authors declare that they have no competing interests.

## Authors' contributions

Both authors conceptualized the study and developed the search strategy and review methodology. CG implemented the search strategy. CG (first reviewer) and PS (second reviewer) both participated in the review of the retrieved articles, and in the interpretation of findings. CG prepared the initial draft of the manuscript. Both authors read and approved the final manuscript

## Pre-publication history

The pre-publication history for this paper can be accessed here:

http://www.biomedcentral.com/1471-2318/9/52/prepub

## Supplementary Material

Additional file 1 **Tables S3 through S6**. Four tables summarizing the studies included in the review. Click here for file
