# Supplementary material for: Comparing the Functional Independence Measure and the interRAI/MDS for use in the functional assessment of older adults: a review of the literature
Source: BMC Geriatr. 2009 Nov 29;9:52. doi: 10.1186/1471-2318-9-52 (PMC2795323; doi:10.1186/1471-2318-9-52)
Supplement: Additional file 1 — Tables S3 through S6. Four tables summarizing the studies included in the review. [file 1471-2318-9-52-S1.pdf]

Table S3: Summary of articles that investigated the reliability of the FIM

<table>
<tr><th>Reference</th><th>Sample and Setting</th><th>Description</th><th>Results</th></tr>
<tr><td>Dallmeijer et al., 2005</td><td>533 participants living independently at home

295 post stroke (mean age 57.5) 150 with MS (mean age 38.3) 88 post TBI (mean age 35.3)</td><td>- Trained Physiatrists assessed all participants with the FIM by direct observation and patient interview
- Investigated internal consistency using Cronbach's alpha
- Separately for each patient group; stroke, MS and TBI
- Considered consistent when alpha >0.70</td><td>- For the patients with stroke, MS and TBI alpha for the FIM motor scale was 0.93, 0.89 and 0.98 respectively
- For the patients with stroke, MS and TBI the alpha for the FIM cognitive scale were 0.78, 0.68 and 0.88 respectively</td></tr>
<tr><td>Daving et al., 2001</td><td>63 stroke survivors living at home

Mean age 53</td><td>- Investigated the reliability of an interview approach for the FIM
- Raters were 3 OTs and 1 nurse trained to use the FIM
- 2 interviews
1) Independent assessments from 2 raters during the same interview in the patient's home
2) Within 1 week on the first interview, independent assessment from 2 raters at the same interview in a clinic
- Interrater reliability at the same and different interviews was assessed using unweighted kappa (wκ, <0.4 poor, 0.41-0.75 good, >0.75 excellent), percentage agreement (PA) (good >80%) and interclass correlation coefficient (ICC) (good > 0.75)</td><td><table>
<tr><td></td><td>Same interview (2 pairs)</td><td></td><td>Different interview (4 pairs)</td><td></td><td>All pairs</td></tr>
<tr><td></td><td>wκ</td><td>PA</td><td>wκ</td><td>PA</td><td>ICC</td></tr>
<tr><td>Motor items</td><td>0.61 – 0.90</td><td>68 – 94</td><td>0.24 – 0.58</td><td>54 – 79</td><td>0.62 – 0.88</td></tr>
<tr><td>Cognitive items</td><td>0.26 – 0.61</td><td>41 – 68</td><td>-0.07 – 0.27</td><td>14 – 46</td><td>0.44 – 0.72</td></tr>
</table>
- Concluded:
- FIM assessment showed high interrater reliability in both settings
- The interrater reliability was lowest when assessment were done at different times by different raters</td></tr>
<tr><td>Dodds et al., 1993</td><td>11,102 patients from 20 inpatient rehabilitation facilities

Mean age 65</td><td>- Each patient was assessed with the FIM by a member of the rehabilitation team at admission and discharge
- Investigated internal consistency using Cronbach's alpha
- Considered consistent when alpha >0.70</td><td>- FIM total at admission $\alpha = 0.93$
- FIM total at discharge $\alpha = 0.95$
- Lower consistency for locomotion subscale (ambulation and stair climbing) 0.68, especially for SCI (0.41) and amputees (0.34)
- Concluded: overall the FIM total has high internal consistency</td></tr>
<tr><td>Fricke et al., 1993</td><td>40 Occupational therapists

4 videos of stroke patients receiving rehabilitation in multiple settings</td><td>- Divided OTs according to experience with the FIM
Experienced = >2 months using the FIM, Inexperienced = <2 months using the FIM
- Randomly assigned the 2 groups to FIM training and non-training groups for a total of 4 groups: experienced and trained, experienced and untrained, inexperienced and trained, inexperienced and untrained
- The trained arm received a 1 hour session including guided instruction, practice and video produced by the UDS
- All participants and one expert (from UDS) rated 4 videos of stoke rehab patients
- Assessed interrater reliability using ICC, percent agreement, disagreement rate and discrepancies b/w the rater and the expert</td><td>1) Percent agreement
Range 57-74%, all activities 65%
<table>
<tr><td></td><td>Trained experienced</td><td>Untrained experienced</td><td>Trained inexperienced</td><td>Untrained inexperience</td></tr>
<tr><td>ICC</td><td>0.89</td><td>0.89</td><td>0.93</td><td>0.80</td></tr>
<tr><td>Percent agreement</td><td>43.5%</td><td>60.5%</td><td>65.1%</td><td>54.7%</td></tr>
<tr><td>Disagreement rate[1]</td><td>0.105</td><td>0.084</td><td>0.076</td><td>0.114</td></tr>
<tr><td>Discrepancies b/w rater and expert[2]</td><td>-0.36</td><td>0.00</td><td>-0.25</td><td>-0.12</td></tr>
</table>
[1]fraction of the distance b/w the expert rating and OT rating, [2]OT rating subtracted from expert rating

Conclusion: "Ratings were most reliable when done by clinicians with no prior experience, from the FIM training group"</td></tr>
<tr><td>Hamilton et al., 1994</td><td>1018 patients from 89 inpatient rehabilitation facilities</td><td>- 2 independent assessment by trained clinicians within 24 hours of admission
- To examine interrater reliability, calculated ICC for FIM total, domains and subscales (one-way random effects ANOVA) and kappa (κ) for individual items (item level, 0.4 good agreement, 0.75 excellent agreement)
- UDS developed a list of criteria for acceptable interrater reliability</td><td>- 24 of the 89 facilities included in the study met all UDS criteria for interrater reliability
- Reported results for entire sample and only for the facilities that met the criteria
<table>
<tr><td></td><td>All Facilities</td><td>Criterion facilities</td></tr>
<tr><td>Total FIM</td><td>ICC = 0.96</td><td>ICC = 0.99</td></tr>
<tr><td>FIM motor
FIM cog</td><td>ICC = 0.96
ICC = 0.91</td><td>ICC = 0.99
ICC = 0.98</td></tr>
<tr><td>FIM subscales</td><td>ICC range = 0.89(Social cognition)-0.94 (Self-care)</td><td>ICC range = 0.97 (sphincter control, locomotion, communication) -0.98 (self-care, transfers, social cognition)</td></tr>
</table></td></tr>
</table>

<table>
<tr><td></td><td></td><td></td><td><table><tr><td>FIM items</td><td>κ range = 0.54 (social interaction) -0.66 (stain climbing)</td><td>κ range = 0.69 (memory) – 0.84 (bladder management)</td></tr></table>Concluded: high interrater reliability when used by clinicians meeting the USD criterion standards, further mastery training and testing in functional assessment seem necessary</td></tr>
<tr><td>Hsueh et al., 1998</td><td>118 inpatients receiving stroke rehabilitation

Mean age 67.5</td><td>- Compared the internal consistency of the FIM motor, the original item Barthel Index (BI) and the BI-5
- Patients were assessed by an OT with both instruments (independently) within 24h of admission and discharge (counterbalanced sequence)
- Used Cronbach's alpha to measure internal consistency (adequate >0.70)</td><td>- Internal consistency was highest for the FIM motor (α = 0.88 - 0.91), and acceptable for all 3 instruments (>0.71)</td></tr>
<tr><td>Jette et al., 2005</td><td>7536 residents from 70 skilled nursing facilities

Mean age 76.3</td><td>- Trained clinicians assessed each patient with the FIM at admission and discharge
- Calculated Cronbach's alpha to investigate internal consistency (considered good where α > 0.70) for each of the 4 FIM domains of functional independence defined by Stineman and colleagues [46] : mobility, ADL, sphincter management and executive function</td><td>- Internal consistency was high for all four domains, ADL α = 0.89, sphincter management α = 0.91, mobility α = 0.76, executive function α = 0.96</td></tr>
<tr><td>Kidd et al., 1995</td><td>25 patients from a neurorehabilitation unit</td><td>- Assessed each patient with the FIM and the BI within 3 days of admission and discharge
- 1st assessment by multidisciplinary team using best available information, 2nd assessment by researcher interviewing each patient, based solely on patient report
- Used the method proposed by Bland and Altman (precision of agreement) to estimate interrater reliability</td><td>Mean difference b/w assessment methods<table><tr><td></td><td>admission</td><td>discharge</td><td>Change</td></tr><tr><td>BI</td><td>0.8 (-4.72-3.12)</td><td>0.44 (-2.02-2.9)</td><td>1.24 (-2.19-4.67)</td></tr><tr><td>FIM</td><td>2.56 (-15.3-10.18)</td><td>0.64(16.8-18.08)</td><td>3.20 (-6.67-13.07)</td></tr></table>- Concluded: variation b/w the two methods that was "proportionately comparable" in both the FIM and the BI</td></tr>
<tr><td>Ottenbacher et al., 1994</td><td>20 community residents receiving assistance from a human service agency

Mean age 75.7</td><td>- Investigated interrater and intrarater reliability of the FIM and IADL of the Multidimensional Functional Assessment of Older Adults
- Assessment model based on the generalizability theory
- Raters were trained members of the research team
- On two occasions, the participants were assessed twice with both instruments, first by the same rater and then by a different rater (total of 4 assessments per participant)
- Applied 2 different testing periods
1) ½ (n=10) short(S) 7-10 days
2) ½ (n=10) long(L) 4-6 weeks
- Estimated reliability using ICC</td><td>ICC values<table><tr><td></td><td>Same rater</td><td></td><td>Different rater</td><td></td></tr><tr><td></td><td>Short interval</td><td>Long interval</td><td>Short interval</td><td>Long interval</td></tr><tr><td>FIM cog.</td><td>.99</td><td>.96</td><td>.99</td><td>.94</td></tr><tr><td>FIM motor</td><td>.97</td><td>.90</td><td>.99</td><td>.91</td></tr><tr><td>FIM total</td><td>.98</td><td>.94</td><td>.99</td><td>.92</td></tr></table>- As expected the ICC was higher for the short time interval than for the longer time interval
- Concluded: the FIM is reliable across raters and overtime</td></tr>
<tr><td>Pollak et al., 1996</td><td>49 residents from a multilevel continuing care retirement community

Mean age 89.7</td><td>- Each participant was evaluated on the FIM twice by a trained researcher
- Testing period of 3 to 8 days
- Used Rasch to converted FIM scores to FIM measures prior to analysis
- Pearson's Correlation Coefficient (PCC) and ICC for repeated measures were used to assess intrarater reliability</td><td>- Found high for the motor (ICC = 0.9) and cognitive (ICC = 0.8) domains
- For higher functioning subjects (SNF), the reliability of the motor subscale (r =0.9) was higher than the cognitive subscale (r =0.6)</td></tr>
<tr><td>Ravaud et al., 1999</td><td>127 patients from a inpatient rehabilitation unit</td><td>- Trained clinicians assessed all patients on admission
- Measured internal consistency of the total FIM using Cronbach's alpha</td><td>- Concluded that the FIM total has high internal consistency (α = 0.93)</td></tr>
<tr><td>Stineman et al., 1996</td><td>93,829 patients discharged from 252 rehabilitation facilities</td><td>- Data provided by the UDS
- Stratified patients by impairment category (using FIM-FRG system), all analysis were done separately for each impairment category
- Examined internal consistency of the FIM total, motor and cognitive; identified items that were highly deterministic of functional status and examined if any items detracted from the overall consistency of the scale/subscale
- Determined item-total correlations with Cronbach's alpha for each item
- Removed items with the lowest item-total correlation to evaluate the effect on the overall consistency of the scale</td><td>- Tub transfer, walking/wheelchair and stairs had low item total correlations in many impairment categories
- No negative item-total correlations
- The highest item-total correlations for the total population were in the mid 80s
- Across the 20 impairment categories – alpha ranged from 0.88 to 0.97 for FIM total, .86-.97 for motor FIM and .86 -.95 for FIM cognitive
- Internal consistency of the total scale/subscale remained generally the same when the lowest correlating item was removed
- Concluded: the FIM has excellent internal consistency, no items should be removed</td></tr>
</table>

Table S4: Summary of articles that investigated the validity of the FIM

Reference	Sample and Setting	Description	Results
Aitken & Bohannon, 2001	28 orthopaedic patients admitted to a subacute setting for rehabilitation<br><br>Mean age 69.1	- Assessed all patients with the FIM and Health-Rated Quality of Life (HRQOL SF-36) within 72 hours of admission and prior to discharge<br>- Also tallied 5 rehabilitation variables (RV, physical therapy visits and units, occupational therapy visits and units and length of stay) for each patient<br>- Estimated the responsiveness of both instruments using Kazis effect size (ES) and t-tests<br>- Examined correlation of both tools to RV using the PCC	- FIM motor, all FIM motor subscales and FIM total were all responsive, with all ES scores between moderate (ES >0.50) and large (ES >0.80)<br>- The FIM cognitive and FIM cognitive subscales were not responsive (ES = 0.09-0.25)<br>- HRQOL SF-36 Physical was moderately responsive (ES = 0.55), all other HRQOL SF-36 subscales were not responsive (ES = 0.03-0.45)<br>- FIM total, motor, self-care and locomotion all correlated (r = -0.403 to -0.692) with all RVs and no other FIM or HRQOL SF-36 measures was correlated with any RVs<br>- Recommended FIM but not HRQOL SF-36 as an outcome measure in subacute rehab. settings
Black et al., 1999	234 stroke patients from an inpatients rehabilitation unit<br><br>Mean age 68.8	- Investigated the relationship between FIM scores at discharge and discharge location<br>- Divided the sample into 2 groups based on discharge location; 1) discharged home, 2) discharged to a skilled nursing facility (SNF)<br>- To dichotomize FIM score, tested multiple cut points to determine which point resulted in the highest number of patients in the expected category (ie high FIM score discharged home and low FIM score discharged to SNF)<br>- Compared the groups with a two-sample median test and chi square statistic	- Found a statistically significant difference between the median FIM admission and discharge scores for the two groups<br>-Discharge FIM scores>80 are associated with discharge to home<br>Sensitivity (0.94) and specificity (0.65)
Brosseau et al., 1995	89 inpatient and outpatient stroke survivors from a neurologic unit<br><br>Mean age 69.8	-Compared 2 alternative FIM administration methods, patient interview (M1) and nurse interview (M2) to the gold standard patient observation (M3)<br>- One physiotherapist was the interviewer/rater for all 3 methods, methods were completed in the same order for each patient and were all completed within a 72 hour period<br>- Dicotomized FIM item scales where 1-5 = no/dependent and 6,7 = yes/independent<br>- Calculated sensitivity, specificity, positive and negative tests for M1 and M2 relative to M3 and ICC between M1 and M2	- M1 and M2 both had high sensitivity (0.79-0.94) and specificity (0.67-1.0) relative to M3<br>- Higher inter-agreement for motor domain (ICC 0.8) than the cognitive domain (ICC 0.64)<br>- Concluded that M3 should not be replaced by M1 or M2, but M1 and M2 are useful alternatives for the motor domain
Brosseau et al., 1996	152 stroke patients in acute hospital care<br><br>Mean age 69	- Every participant was assessed on the FIM, Fugl-Meyer Test (motor status) and with an assessment tool published by the Ontario Society of Occupational Therapy (OSOT, cognitive status) by a trained PT within 72h of admission<br>- Used a factor analysis to examine the dimensions included in the FIM (Principal Component Analysis, PCA)<br>- Compared PCC to examine the association between the FIM motor and FIM cognitive with the Fugl-Meyer and the OSOT respectively	- 2 factors were found: life habits/ADL (FIM motor) and neuropsychological ability (FIM cog.)<br>- They accounted for 76.2% of the total variance<br>- The FIM motor and FIM cognitive showed positive association with the Fugl-Meyer and the OSOT respectively<br>- Concluded: the FIM has a bidimentional structure<br>- Concluded: FIM motor could be used alone or in combination with the FIM cognitive for clinical prediction purposes
Bunch & Dvonck, 1994	Conjoint analysis: 58 rehabilitation team members<br><br>Multiple regression:142 patients receiving hip fracture rehabilitation	- Used conjoint analysis and multiple regression to investigate the equivalence (desirability) of the FIM subsections and assess the implications on the meaning of the FIM total score<br>- Assessed the significance by contrasting the regression equation developed by the conjoint analysis with the regression equation developed with a sample of hip fracture patients	- 12% range in desirability across the 4 subsections tested<br>- continence and mobility had uniform spacing (interval)<br>- self care and communication were not linearly related (not interval)<br>- The two regression equations produced the same result<br>- Concluded: when other sources of error are considered the difference in desirability between the subsections is very small, and in practice FIM items can be summed to a meaningful total score (interval)
Cano et al., 2006	1,495 MS, stroke and SCI patients from a neurorehabilitation unit<br><br>Mean age 48	- Assessed all participants with the FIM motor and the BI within 3 days of admission and 2 days of discharge<br>- Examined item and total score distributions on admission and discharge<br>- Assessed responsiveness of the items and total score using ES	Total scores<br>- Ceiling effects were lower for the FIM motor than the BI (adm. = 0.4/1.7 and dis. = 5.4/27.8)<br>- ES were similar for both measures (FIM = 0.74, BI = 0.77)<br>Item scores<br>- Floor and ceiling effects were lower for all FIM items than comparable BI items<br>- ES was higher for 2 BI items (Feeding, bathing) and 2 FIM items (bowels, walk/wheelchair

<table>
<tr><td></td><td></td><td></td><td>use) equal for 4 items (grooming, toileting, bladder, stairs); FIM ES = 0.27-0.82, BI ES = 0.20 – 0.80
Concluded: the BI and the FIM are equally responsive to clinically relevant change</td></tr>
<tr><td>Cotter et al., 2002</td><td>21 participants with dementia living at home and their primary caregiver

Mean age 62</td><td>- Aim was to determine if caregivers of dementia patients can validly report the patients ADL dependence and time spent providing ADL assistance
- Caregivers assessed patients using the 6 FIM self-care subscale items and 1 mobility item (bed/chair/wheelchair transfer) (caregiver-reported, CR) and reported how much time in minutes was spent assisting with each ADL item
- ADL performance was then videotaped in the home and two independent trained raters assessed the patient's functional status using the same FIM items (observation derived, OD)
- Correlation b/w the FIM scores was examined using Sperman's rho and Wilcoxon signed-ranks
- Correlation b/w the caregiver's time estimate and the observed time from the videotape were examined using PCC and Wilcoxon signed-ranks</td><td>- The correlation of functional status b/w the CR and OD ranged from 0.620 and 0.909, and for 6 of the 7 items there was no statistically significant difference
-There was a statistically significant difference b/w CR and OD for the transferring item (p = 0.0014)
- According to the Wilcoxon signed ranks, all CR time estimates were larger than the OD assistance durations; with bathing, dressing below the waist and toileting being statistically different
- On average the CR estimates were 2-3 times greater than the OD assistance durations
- Concluded: caregivers can report the nature of their ADL assistance with reasonable accuracy, however due to consistent overestimates should but used with caution</td></tr>
<tr><td>Cotter et al., 2008</td><td>21 participants with dementia living at home and their primary caregiver

Mean age: 62</td><td>- Aim was to determine if caregivers of dementia patients can validly report functional status
- Caregivers assessed patients using 6 FIM self-care items and 1 mobility item (bed/chair/wheelchair transfer) (caregiver-reported, CG)
- ADL performance was then videotaped in the home and two independent trained raters assessed the patient's functional status using the same FIM items (observation derived, OD)
- The videotaped data were then assessed by a blinded, trained OT (OT-rated, OT)
- Correlation b/w the FIM scores was examined using Sperman's rho (b/w 3 sets), ANOVA for overall FIM difference, and t-tests for each ADL item</td><td>- All correlations were positive and statistically significant at p<0.005 or better
<table>
<tr><td>ADL</td><td>CG/OD</td><td>CG/OT</td><td>OD/OT</td></tr>
<tr><td>Bathing</td><td>0.904</td><td>0.884</td><td>0.924</td></tr>
<tr><td>Dressing above waist</td><td>0.910</td><td>0.891</td><td>0.893</td></tr>
<tr><td>Dressing below waist</td><td>0.818</td><td>0.915</td><td>0.933</td></tr>
<tr><td>Eating</td><td>0.862</td><td>0.717</td><td>0.809</td></tr>
<tr><td>Grooming</td><td>0.620</td><td>0.860</td><td>0.862</td></tr>
<tr><td>Toileting</td><td>0.858</td><td>0.795</td><td>0.909</td></tr>
<tr><td>Transferring</td><td>0.700</td><td>0.891</td><td>0.764</td></tr>
</table>
- Concluded: caregiver ratings are comparable to those obtained from a trained evaluator and caregivers can accurately describe the ADL functioning of their loved ones with dementia</td></tr>
<tr><td>Dallmeijer et al., 2005</td><td>533 participants living independently at home

295 post stroke (mean age 57.5) 150 with MS (mean age 38.3) 88 post TBI (mean age 35.3)</td><td>- Trained Physiatrists collected FIM score by direct observation and patient interview
-Factor analysis
- PCA follow by orthogonal rotation
- Item considered to load on a factor if the factor loading was higher than 0.40
- Separately for each the motor and cognitive domain and each impairment group
- Rasch analysis
- First analysed pooled data to assess item fit in each domain
- Second, examined differential item functioning (DIF) between impairment groups
- To determine items difficulties are the same across the 3 impairment groups</td><td>- Low number of responses in the dependent categories
- condensed number of item response categories from 7 to 3 where 1-5 = 1, 6 = 2 and 7 = 3
- In all groups found two factors, motor and cognitive
- the motor factor accounted for 47%, 39% and 54% of the total variance in stroke, MS and TBI groups respectively
- the cognitive factor explained 18%, 17% and 23% in the stroke, MS and TBI groups respectively
- Concluded: the FIM has a 2 dimensional structure
Rasch
- pooled data: 2 misfit items in motor domain, bowel and bladder, removed for DIF
- DIF was found in 7 of the 11 motor items and 4 of the 5 cognitive items
- Concluded: there is limited comparability across impairment groups, must only be performed after adjustment for DIF</td></tr>
<tr><td>Desrosiers et al., 2003</td><td>132 post stroke patients from an inpatient rehabilitation unit

Mean age 69.9</td><td>- Compared the association and responsiveness of the Functional Autonomy Measurement System (SMAF) and the FIM and the association of each instrument to a social participation measure after rehabilitation
- All participants were assessed with the SMAF and the FIM on admission, 2 weeks post discharge and 6 months post discharge
- At both post discharge assessments, the assessment of life habits (LIFE-H) was also administered
- Calculated PCC to investigate association between scales, the relationships to the LIFE-H were further investigated with the method described by Meng and colleagues [61]</td><td>- There were moderate to strong relationships (alpha 0.65-0.96) between corresponding categories of the FIM and the SMAF
<table>
<tr><td>FIM items</td><td>SMR</td><td>SMAF items</td><td>SMR</td></tr>
<tr><td>Self care + sphincter control</td><td>0.77</td><td>ADL</td><td>0.88</td></tr>
<tr><td>Mobility + locomotion</td><td>1.54</td><td>Mobility</td><td>1.28</td></tr>
<tr><td>Communication</td><td>0.06</td><td>Communication</td><td>0.09</td></tr>
<tr><td>Social cognition</td><td>0.05</td><td>Mental function</td><td>0.08</td></tr>
<tr><td>-</td><td>-</td><td>IADL</td><td>0.97</td></tr>
<tr><td>Total score</td><td>0.97</td><td>Total score</td><td>1.20</td></tr>
<tr><td></td><td>-</td><td>Total score IADL</td><td>1.04</td></tr>
</table></td></tr>
</table>

<table>
<tr><td></td><td></td><td>- Responsiveness was measured using paired t-tests and SRM</td><td>- All corresponding FIM and SMAF categories were equally responsive with the exception of the SMAF total score that was significantly more responsive than the total FIM
- The total SMAF and FIM are both highly related to the total LIFE-H, also corresponding components of the FIM and SMAF follow similar patterns of correlation to the LIFE-H components
- 3 LIFE-H domains (education/employment, leisure and interpersonal relationships) were not related to either the SMAF or the FIM</td></tr>
<tr><td>Dickson & Kohler, 1995</td><td>515 patients from an inpatient rehabilitation unit

2 subgroups within the total group; 313 with neurological disorders and 41 with amputations</td><td>- Investigated the dimensionality of the FIM items
- Correlation matrix of all FIM motor items
- Calculated PCC
- Factor analysis, PCA analysis to investigate the dimensional structure of the FIM motor
- Eigenvalues >1 were used to identify factors</td><td>- Magnitude of correlation not consistent b/w items
- strong correlations b/w transfer items, poor correlation b/w stair climbing and eating
- For all 3 analyses more than one factor was required to explain the variance

Percent Variance per Factor<table>
<tr><td></td><td>1</td><td>2</td><td>3</td><td>4</td><td>5</td><td>6</td></tr>
<tr><td>Total pop.</td><td>63.886</td><td>10.137</td><td>6.959</td><td>4.038</td><td>3.310</td><td>2.767</td></tr>
<tr><td>Neuro.</td><td>67.124</td><td>11.097</td><td>6.096</td><td>3.286</td><td>2.872</td><td>2.388</td></tr>
<tr><td>Amputee</td><td>58.508</td><td>9.998</td><td>6.877</td><td>6.200</td><td>4.294</td><td>3.912</td></tr>
</table>- For all patients and the group of patients with neurological conditions, 3 factor were required to explain 80% of the variance
- For the group of patients with amputations, 4 factors were required to explain 80% of the variance
- Concluded: the FIM total and FIM motor are both not unidimentional, therefore it is not appropriate to use Rasch analysis</td></tr>
<tr><td>Dodds et al., 1998</td><td>11,102 patients from a inpatient rehabilitation unit

Mean age 65</td><td>- Developed multiple hypothesise to test the FIM's ability to discriminate between patient characteristics and impairment type
- Each patient was assessed with the FIM at admission and discharge
-Also investigated responsiveness with paired t-tests</td><td>- All hypothesise were confirmed
- Concluded: FIM is able to discriminate between impairment types
- On average the patients showed 33% (6 points) improvement ($p < 0.001$) on the FIM
- Responsiveness differed among types of impairments
- Concluded: the FIM may be a responsive measure
- Author notes interpretation of change scores is not clear and calls for further examination</td></tr>
<tr><td>Gosman-Hedstrom & Svensson, 2000</td><td>204 participants 3 months post-stroke in multiple settings

Age 70+</td><td>- Each participant was assessed with the FIM and the BI by an OT
- FIM items were condensed to 2-4 response options to correspond to the BI
- Used rank invariant statistical method (item level comparison) to estimate correlation between the FIM and BI</td><td>- High concordance b/w FIM and BI
- Monotonic agreement (0.978-1), percent agreement (0.62-0.97)</td></tr>
<tr><td>Granger et al., 1986</td><td>114 clinicians evaluated 110 rehabilitation patients</td><td>- Pilot test for face validity
- An average of 3.5 clinicians partially assessed each participant
- Raters from a wide variety of areas: OT, PT, nurses, doctors, speech pathologists, recreational therapists, social workers, researchers
- After the rater assessed the patient they were asked
1) Are any items difficult to understand?
2) Are there any unnecessary items?
3) Should any items be added?
- Also asked to rate the FIM on a 5 point global scale with respect to its adequacy as a measure of severity of disability (1 = poor – 5 = excellent)</td><td>- 12% of the raters agreed with the first question
- item wording was revised
- the number of response options was increased from 4 to 7
- modified dependence was segmented into supervision, minimal assistance and moderate assistance
- Only 0.3% agreed with the second question, no items were eliminated
- 30.7% agreed with the third question
- 2 items were added
- The average rating on the global scale was 3.2 (SD 0.55)</td></tr>
</table>

Granger et al., 1993a	21 participants discharged from inpatient rehabilitation (living in the community)<br><br>Mean age 65.9	- Investigated whether FIM scores are able to predict: burden of care (minutes of care provided in the home/day), and subjects life satisfaction<br>- Patient (or family member) completed a "Help at Home Journal", recorded actual help received per day<br>- Researcher (trained to administer the FIM) assessed patients at home by interview and patient observation<br>- Selected specific items from other functional assessment scales including; Environmental Status Scale (ESS), Incapacity Status Scale (ISS), Long-range Evolution System (LES), Brief Symptom Inventory (BSI), Sickness Impact Profile (SIP), and assessed all patient with selected items<br>- Calculated the PCC for each item, subscale, domain and full scale with the two dependent variables<br>- Conducted simple regression, and multiple regression analyses (using the step-wise method) to determine the contribution of each item, subscale, domain and full scale to predicting the two dependent variables	- The FIM total and FIM motor scores showed high negative correlation with help received per day (PCC = -0.79 and PCC = -0.81 respectively), however did not correlate with general life satisfaction<br><br>Multiple regression (R^2)<br>Help in minutes/day<br>FIMTUB, FIMGRM, FIMLOCOMO, FIMDRLO,SIPPHYS,FIMCPHM — .73, .89, .92, .95, .97, .98<br>General life satisfaction<br>BSIDEP, FIMDRLO, ISSVSN, FIMCOG, BSIHOS — .54, .75, .83, .91, .95<br>- Concluded: the FIM and the SIP are both useful in predicting burden of care and the FIM contributed to predicting the level of life satisfaction<br>- A one point change on the FIM 18 and FIM 13 was equal to 2.19 and 2.37 minutes of help respectively
Granger, 1993b	REANALYSED Heinemann et al., [65] And Linacre et al., [66]	- Investigated operating characteristics and DIF of the FIM using Rasch analysis<br>- First analysed the full data set as a single population, then analysed separately for USD defined impairment groups	- Found 2 dominant patterns of difficulty; motor and cognitive subscales<br>- Easiest and hardest motor items are eating and stair climbing respectively<br>- Easiest and hardest cog. items are expression and problem solving respectively<br>- Major patterns were consistent across impairment groups, with few expected exceptions based on patient characteristics<br>- For the same change in FIM score (ordinal), the change in FIM measure (interval) is less in the middle of the scale than at the top and bottom end
Grimby et al., 1996	579 patients from rehabilitation medicine wards in 3 hospitals<br><br>Mean age 45	- All participants were assessed by a rehabilitation team (physician, nurse, OT and PT) within 1 week of admission and 1 week of discharge<br>- Divided the patients into 6 diagnostic groups<br>- Used Rasch to analyse possible DIF for the diagnostic groups and b/w admission and discharge assessments<br>- Performed a multiple regression analysis with length of stay as the dependent variable and age, sex, FIM data and changes from admission to discharge as independent variables	- Bowel and eating were the easiest motor items and stairs was the most difficult<br>- For motor items, found good agreement between the diagnostic groups with few differences that could be explained by diagnostic characteristics<br>- Memory and problem solving were the hardest among the cognitive items<br>- The cognitive items were more "diagnosis sensitive" than the motor items<br>- Found a sigmoidal relationship between FIM raw scores and FIM measures<br>- Admission FIM accounted for up to nearly 50% of the variation in length of stay in a homogeneous sample such as stroke patients, but less than 40% in the total sample.<br>- Concluded that FIM data can be used for comparison of patient status at admission and discharge in different rehabilitation units
Heinemann et al., 1993	27,669 rehabilitation patients<br><br>Mean age 62.1	- Used Rasch to convert FIM scores to FIM measures and examine DIF across impairments groups<br>- Hypothesis: the items on the FIM motor and cognitive domains each form one unidimentional scale with item difficulties being consistent across groups<br>- Assessment by trained clinicians within 72 hours of admission and discharge<br>- Analysed the motor and cognitive domains separately<br>- Examined data for the entire sample and then separately for each of the 13 impairment groups (USD definitions)	- For the motor domain feeding and grooming were the easiest items, stair climbing, locomotion and tub/shower transfer are the most difficult items<br>- For the cognitive domain comprehension and expression were the easiest items and problem solving was the most difficult<br>- All items had acceptable fit, however in the motor domain bowel, bladder and stair fit less well<br>- PCA showed that 95 and 92 percent of the variance is explained by the model for the motor and cognitive domains respectively<br>- Item functioning was relatively equal across impairment groups, there were few exceptions that paralleled impairment characteristics<br>- Specific suggestions for improving the FIM<br>- reduce the number of transfer items, partially redundant<br>- separate the bowel and bladder items further to distinguish cause for the incontinence<br>- 3 items that have 2 modes – locomotion (wheelchair vs walking), comprehension (auditory vs visual) and expression (vocal vs nonvocal) and could read as separate items<br>- develop an easier stair climbing item<br>- "not tested" items could be assigned a value other than 1 to distinguish them from "total dependence"<br>-Concluded: raw scores are not linear and should not be used in parametric statistical analysis

<table>
<tr><td>Heinemann et al., 1994</td><td>27,600 patients from 72 inpatient rehabilitation facilities

Mean age 62.1 years</td><td>- Evaluated the extent to which functional status measures can be used to predict rehabilitation outcome and resource use
- Hypothesis: 1) functional status at discharge could be predicted by admission function and 2) length of stay (LOS) could be predicted by admission functional status and promptness of admission following impairment
- Assessment by trained clinicians within 72 hours of admission and discharge
- Patients were separated by UDS-defined impairment types
- Used Rasch to convert FIM scores to FIM measures prior to analysis
- Performed multiple logistic regression with the clinical features (FIM scores and other patient characteristics determined by previous literature review) as the independent variables and discharge motor function, discharge cognitive function and LOS as the separate dependent variables</td><td><table>
<tr><th></th><th>Percent variance (range for impairment types)</th><th>Most powerful predictor</th><th>Other significant predictors</th></tr>
<tr><td>Discharge motor function</td><td>55 (47-71)</td><td>FIMmotor on admission</td><td>Rehabilitation interruptions and onset admission interval</td></tr>
<tr><td>Discharge cognitive function</td><td>70 (46-85)</td><td>FIMcog. on admission</td><td>Age, promptness of admission and frequency of rehabilitation interruptions</td></tr>
<tr><td>LOS</td><td>20(6-36)</td><td>FIM motor on admission</td><td>Age, promptness of admission, cognitive function and frequency of rehabilitation interruptions</td></tr>
</table>
- Admission functional status was consistently related to discharge function and LOS
- Motor function was a more important predictor of LOS than cognitive function for all impairment groups
- Concluded: FIM should be used in the development of rehabilitation resource use models</td></tr>
<tr><td>Hsueh et al., 1998</td><td>118 stroke rehabilitation inpatients

Mean age 67.5</td><td>- Investigated the concurrent criterion validity and responsiveness of the FIM motor, BI and BI-5
- Patients were assessed by an OT with both instruments (independently) within 24h of admission and discharge (counterbalanced sequence)
- Examined score ranges to assess floor and ceiling effects
- Transformed both scales to range 0-100 by: 100* (observed score-minimum possible score)/score range
- Used Spearman's Correlation Coefficient and ICC to investigate the interaction across the measures (where ICC>0.75 indicated excellent agreement)
- To measure responsiveness calculated the standardized response mean (SRM)
    - used Cohen's criteria, >0.8 is large, 0.5-0.8 is moderate and 0.2-0.5 is small
    - used Wilcoxon matched pairs to evaluate significance</td><td>- B1-5 had significant floor effects at admission, 46.6% of the sample was in the floor

Correlation with the FIM at admission and discharge
<table>
<tr><th></th><th>Admission (r, ICC)</th><th>Discharge (r, ICC)</th></tr>
<tr><td>BI-10</td><td>0.92, 0.83</td><td>0.94, 0.87</td></tr>
<tr><td>BI-5</td><td>0.74, 0.36</td><td>0.94, 0.74</td></tr>
</table>
Responsiveness
<table>
<tr><th></th><th>SRM</th><th>Wilcoxon Z (p value)</th></tr>
<tr><td>FIM</td><td>1.3</td><td>7.5 (<0.001)</td></tr>
<tr><td>BI-10</td><td>1.2</td><td>7.4(<0.001)</td></tr>
<tr><td>BI-5</td><td>1.2</td><td>7.0 (<0.001)</td></tr>
</table>
- Concluded: BI and FIM both have acceptable and similar psychometric properties</td></tr>
<tr><td>Jette et al., 2005</td><td>7536 residents from 70 skilled nursing facilities

Mean age 76.3</td><td>- Investigated the validity of using FIM items to derive 4 domains of functional independence defined by Stineman and colleagues [46] : mobility, ADL, sphincter management and executive function
- Trained raters assessed each patient with the FIM at admission and discharge
- Factor analysis (PCA)
- Separately for each domain conducted an item level analysis (mean, SD, skewness), corrected item-total correlations (>0.40 considered good correlation), and floor and ceiling effects</td><td>- Only 4 factors had eigenvalues above 1, the four factors accounted for 73.4% of the variance in functional independence
- The items in each domain had similar SDs and distributions of items were not highly skewed
- The item total correlations were higher within each domain than with items outside each domain
- At admission, there were floor effects for sphincter management (34.4%) and mobility domains (43.1%) and ceiling effects for executive function domain (26.7%)
- Concluded that the 4 FIM domains described by Stineman and colleagues [46] are valid for describing the functional independence of SNF residents</td></tr>
<tr><td>Kidd et al., 1995</td><td>25 patients from a neurorehabilitation unit</td><td>- All patients were independently assessed with the FIM and the BI by a multidisciplinary team within 3 days of admission and discharge
- Converted FIM scores (ordinal) to FIM measures (interval, 0-100)
- Dichotomized total FIM measures and BI scores at midpoint and constructed a 2-by-2 table to assess agreement
- Calculated unweighted κ to measure the degree of agreement</td><td>- 14% of the items changed on the FIM, but did not change on the BI, 2% of the items changed on the BI but not on the FIM and 33% changed on both tools
- κ: admission 0.92 (CI 0.77-1.0), discharge 0.88 (CI 0.66-1.0), change 0.78 (CI 0.49-1.0)
- Concluded: there is reasonable agreement b/w the measures at admission and discharge, and only moderate agreement of change; the FIM has no psychometric advantages over the BI</td></tr>
<tr><td>Linacre et al., 1994</td><td>14,799 patients from a inpatient rehabilitation unit</td><td>- Obtain admission and discharge FIM ratings from the UDS_{MR}
- Used Rasch analysis to convert FIM ordinal scores to interval measures
- Analysed the dimensionality of the FIM
    - assessing fit statistics(Fisherian acceptance testing)
- Examined DIF between admission and discharge measures to establish whether it is appropriate to use the FIM to measure change over time</td><td>- Initial analysis on all 18 items
    - easiest item = eating, hardest item = stair
    - 2 of 5 cognitive item misfit
    - all fit stat. for cognitive items above 1, fit stat. for 8 of 13 motor items below 1
        - Concluded: evidence of multidimensionality
- Separated motor and cognitive items and re-analysed data
    - all cognitive items had acceptable fit</td></tr>
</table>

			- 4 motor items did not fit the model; bowel, bladder, stair, eating<br>- eating and stair are the easiest and hardest items, therefore most likely to misfit<br>- bowel and bladder likely physical and neurological components<br>- range of item calibrations was greater when domains were separated, evidence that the tool is more discriminative<br>- Concluded: 1) neither FIM motor or cognitive scores are linear (S shaped curves),2) there are slight differences in how tool functions at admission and discharge, however these are small enough to not be clinically relevant, therefore the FIM can be used to measure change over time
Lundgren-Nilsson et al., 2005a	1660 patients with stroke, TBI, SCI admitted to inpatient rehabilitation facilitates<br><br>Mean age 48	- All participants were assess with the FIM by a trained rater on admission<br>- Analysed the structural properties of the FIM's response options using Rasch analysis (ie. investigated disordered thresholds)<br>- Examined category probability curves for evidence of disordered thresholds, where necessary collapsed categories to determine the best model based on: person separation, disordered categories, distance of more than 1.4 logits between categories and item fit to the model	- For all 3 diagnosis, disordered thresholds were present when all 7 response options were used<br>- A scale with 4 response options (complete dependence, modified independence, partial dependence, total dependence) is the best fit solution for all 3 diagnosis
Lundgren-Nilsson et al., 2005b	2546 inpatients from 31 rehabilitation facilities within 6 different European countries<br><br>Mean age 62	- Aimed to analyse the cross cultural validity of the FIM using the Rasch model<br>- Initially data from each country was analysed separately and then pooled to assess cross-cultural differences<br>- Examined output for disordered thresholds and collapsed middle categories uniquely for each item and country<br>- Refit collapsed categories to the Rasch model for each country using standardized fit statistics for persons and items (acceptable range +/- 3.0) and a chi-square item-trait interaction statistic (non significant chi-square, >0.05)<br>- DIF analysis within each country for age and gender, DIF analysis on pooled data for country<br>- PCA of fit data to assess dimensionality	- Disordered thresholds were found especially for toileting, bladder and bowel management, transfer tub/shower, walk/wheelchair and stairs<br>- In all countries there were few disordered thresholds in the FIM cognitive<br>- Eating was the easiest item and transfer tub/shower and stairs were the most difficult items in most countries<br>- Fit to the Rasch model varied by country for the motor scale, items fit the model in UK and item fit sequentially decreased in France, Belgium, Italy, Israel and Sweden respectively<br>- FIM cognitive items fit the model in every country except Israel<br>- The refit motor and cognitive scales for the individual countries were all free of DIF by gender and all but Sweden were free of DIF by age<br>- In the pooled data only 5 of the 13 motor items had ordered thresholds, after collapsing the number of response categories varied from 2 to 7 across the items<br>- Expression was the only cognitive item that had disordered thresholds in the pooled data<br>- 7 of the motor items and 1 of the cognitive items showed DIF by country<br>- After adjusting for DIF by country the pooled data fit the Rasch model<br>- Concluded: FIM data for patients with stroke cannot be pooled in its raw form, or compared across countries; comparisons can only be made after adjusting for country specific DIF
Lundgren-Nilsson et al., 2006	471 patients from 9 inpatient rehabilitation facilities<br><br>Age range 11-90	- Used Rasch techniques to investigate validity of the FIM<br>- item response options: examine output for disordered thresholds and collapsed middle categories where necessary<br>- item fit: positive residuals above 2.5 were considered to fit the model<br>- DIF: b/w diagnostic group (stroke, TBI, SCI), used Tukey's post hoc tests to determine where the DIF occurred when more than 2 groups were compared<br>- Analysed the clinical meaning of the DIF<br>- used a test equating technique to determine whether the meaning of the sum score reflected the same amount of independence in each group<br>- used boundaries set by Lai and Eton [72]	- Item response options<br>- For separate group data and pooled data, disordered thresholds were found for the majority of items<br>- most item response options were reduced to 3 categories where, new 1 (old 1, 2) new 2 (old 3, 4, 5) new 3 (old 6, 7)<br>- in the separate group data, SCI grooming and stairs were dichotomised, for TBI stairs was dichotomised<br>- in the pooled data bladder and stairs were dichotomized<br>- Item fit<br>- Fit was assessed on re-scaled data<br>- In the separate group data items, all items in the stroke and TBI group fit the model, in SCI bladder and bowel misfit<br>- In the pooled data eating and bowel misfit the model<br>- DIF<br>- pooled data from all 3 groups was analysed for DIF, all items had DIF<br>- Turkey's post hoc showed that 9 of the 13 had DIF for SCI<br>- due to the large amount of DIF items, SCI group was removed from the pooled data for further analysis

<table>
<tr><td></td><td></td><td></td><td>- there were no misfit items in the pooled data when the SCI group was removed
- DIF with SCI removed
- 6 of the 13 items showed DIF
- the scale was split for DIF items, making a new scale of 19 items
- Analysis of the clinical significance of the DIF showed no clinical relevance
- Concluded: number of item response options should be reduced, suggested that the reason for the DIF having no clinical relevance is that for the sum score the individual item's DIF "balance out" - calls for further examination, SCI patients are different from stroke and TBI patients (not the same construct)</td></tr>
<tr><td>Oczkowski & Barreca, 1993</td><td>113 patients from a stroke-specific rehabilitation program

Mean age 65.7</td><td>- Investigate the potential of the FIM as a prognostic indicator of outcome
- All patients were assessed on the FIM by a multidisciplinary team within 1 week of admission and then biweekly
- Performed multiple logistic regression with the clinical features (demographic information, neurological characteristics, length of time from stroke onset, FIM scores) as the independent variables and the discharge location as the dependent variable</td><td>- Bladder and bowel incontinence on admission were predictive of discharge location
- Gender, side of paralysis, hemianopsia, neglect, depression, aphasia and motivation were not predictive of discharge location
- FIM scores on admission was the most powerful predictor of discharge location, admission postural staging and age were also significant predictors
- Patients with admission FIM scores of 36 or less were never sent home, whereas all patient with FIM admission scores over 97 were discharged home
- Concluded: It is possibly to use the FIM to classify stroke patients according to their needs</td></tr>
<tr><td>Ottenbacher et al., 1994</td><td>20 community residents
receiving assistance from a human service agency

Mean age 75.7</td><td>- Investigated association between the FIM and the Multidimensional Functional Assessment of Older Adults IADL scale
- Raters were trained members of the research team
- On two occasions the participants were assessed twice on both measures, first by the same rater and then by a different rater (total of 4 assessments per participant)
- Calculate PCC to measure association</td><td>- The instruments were strongly correlated when both instruments were administered by the same rater (PCC=0.87) and when administered by different raters (PCC=0.83)</td></tr>
<tr><td>Pollak et al., 1996</td><td>49 residents from a multilevel continuing care retirement community

Mean age 89.7</td><td>- Group subjects according to care setting: independent community, sheltered care, or skilled nursing facility
- Each participant was evaluated twice by a trained researcher, 3 to 8 days between assessments
- Rasch analysis was used, separately for the motor and cognitive domains, to converted FIM scores to FIM measures
- Assessed item difficulties and fit statistics
- Compared item difficulty calibrations (logits) found in this study with the those obtained by Linacre and colleagues [66]
- Used two one way ANOVA to investigate the difference between the 3 groups</td><td>- The motor and cognitive domains are both unidimensional, linear scales
- Eating and stair climbing were the easiest and hardest items on the motor domain respectively, and expression and problem solving were the easiest and hardest items on the cognitive domain
- 3 misfit items on the motor scale: bladder management, bowel management and grooming, 1 misfit item on the cognitive subscale: memory
- Significance difference between residential groups for both the motor (F (34.71), p<.05) and cognitive (F(12.42), p<.05) domains, provides evidence that the FIM measures level of assistance
- Correlation of item with Linacre [66]: high for the motor subscale (r = 0.9) and low for the cognitive subscale (r = -0.3)
- Suggests this is due to different populations</td></tr>
<tr><td>Ravaud et al., 1999</td><td>127 patients from a inpatient rehabilitation unit</td><td>- Trainer clinicians assessed all participants on admission
- Analysed variable interdependence by constructing a correlation matrix b/w all individual items (Persons Correlation Coefficient)
- Reasoned that if they are all measuring the same construct that they should all correlate with an alpha of at least 0.45
- Factor analysis to investigate the dimensionality of the FIM
- PCA, analysed output before and after an orthogonal transformation</td><td>- Found that many of the items did not correlate with at least 0.45
- comprehension and expression show the lowest correlation with the other FIM items
- results suggest that motor items involving limbs are independent of the cognitive items
- PCA, before rotation
- 2 factors, motor and cognitive domains, explain 63.7% of the variance
- PCA, after orthogonal rotation
- 4 factors (explained 76.5% of the variance)
- F1 mobility and locomotion, F2 cognitive items, F3 self care items, F4 sphincter items
- Conclusion: "neither the FIM nor the motor subscore are unidimensional</td></tr>
<tr><td>Schepers et al., 2006</td><td>163 post-stroke patients admitted to inpatient rehabilitation units

Mean age 56</td><td>- Compared the responsiveness of several instruments used in stroke research: BI, FIM, Frenchay Activities Index (FAI) and Stroke Adapted Sickness Impact Profile 30 (SA-SIP 30)
- All patients were assessed with the BI and the FIM at admission, 6 months (subacute phase, SP) and one year post stroke (chronic phase, CP), and assessed with the SA-SIP 30 and FAI at 6 months and one year post stroke
- Responsiveness was measured using ES (small 0.2-0.5, moderate 0.5-0.8, large >0.8)</td><td>Effect size<table>
<tr><td></td><td>SP</td><td>CP</td><td></td><td>SP</td><td>CP</td></tr>
<tr><td>BI</td><td>0.98</td><td>0.52</td><td>SA-SIP30 total</td><td>-</td><td>0.63</td></tr>
<tr><td>FIM total</td><td>0.84</td><td>0.47</td><td>SA-SIP30 physical</td><td>-</td><td>0.53</td></tr>
<tr><td>FIM motor</td><td>0.89</td><td>0.51</td><td>SA-SIP30 psychological</td><td>-</td><td>0.64</td></tr>
<tr><td>FIM cognitive</td><td>0.47</td><td>0.47</td><td>FAI</td><td>-</td><td>0.59</td></tr>
</table>- Concluded: the BI, FIM total, FIM motor, FAI, SA-SIP30 are responsive measures and</td></tr>
</table>

			recommend using the BI in the SP and the FAI and SA-SIP in the CP
Stineman et al., 1996	93,829 patients discharged from 252 rehabilitation facilities	- Data provided by the UDS<br>- Stratified patients by impairment category of the latest FIM-FRG system, 20 impairment categories, all analysis were done separately for each impairment category<br>- Investigated the distribution of item-level responses and assessed whether any response options or items could be removed to improve the psychometric properties of the FIM but maintain clinical utility<br>- looked for unused item response options and items that all participants responded the same<br>- tallied the distribution of item responses in all 20 impairment groups<br>- investigated floor and ceiling effects by identifying items that have an average response less than 3 or greater than 5, and items that have an average response that were greater or less than the means of all items by more than 2 SD<br>- Factor analysis of explore 2 dimensional structure<br>- PCC (orthogonal rotation), forced 2 factor solution<br>- items were considered to belong to the factor were it had the highest loading, if it had a loading above .4 on both factors it was considered multidimentional<br>- Multitrait scaling analysis to assess validity of the summation of the motor and cognitive domains<br>- predetermined a series of 5 situations to validate the FIM as motor and cognitive summated subscales	-Analysis of item-level responses<br>- consistent finding across all impairments<br>- all response options were used for every item<br>- item 6 “modified independence” was chosen less frequently then item 7 “total independence”, however found no psychometric benefit results from collapsing item 6 and 7, suggest they remain separate for clinical meaning<br>- found no ceiling effects<br>- in 3 of the impairment groups “stair” had a floor effect<br>- Factor analysis<br>- in 16 of the 20 impairment groups the items factored on to the motor and cognitive domains<br>- in the 4 remaining groups 6 or less items were multidimentional<br>- Multitrait scaling<br>- “Overall results support expression of the motor and cognitive FIM subscales as summated ratings”
Stineman et al., 1997	93,829 patients discharged from 252 rehabilitation facilities	- Data provided by the UDS<br>- Stratified patients by impairment category of the latest FIM-FRG system, 20 impairment categories, all analysis were done separately for each impairment category<br>- Factor analysis to investigate the existence of finer factors within the motor and cognitive domains<br>- PCA (orthogonal rotation), did not numerically limit the factor solution<br>- items were considered to belong to the factor where it had the highest loading<br>- If/when impairment specific factors were identified, Cronbach’s alpha was calculated to determine internal consistency of the factor	- In 18 of the 20 impairment categories impairment specific structure was found beyond the motor dimension<br>- The additional factors were always nested in the motor dimension, the cognitive factor remained undivided in all cases<br>- 2 impairment categories loaded on 2 factors (motor and cognitive domain), 4 had 3 factors, 14 had 4 factors<br>- The most common new factors found were; 16 categories had a mobility dimension (mobility and locomotion subsets), 13 had a self care dimension, 13 had a sphincter control dimension, 3 had an ADL dimension (self care + sphincter control)<br>- Conclusion: there are multiple ways to divide the FIM into subscales, the appropriate subscale to use is dictated by the research question
Streppel & Van Harten, 2002	48 stroke patients at inpatient rehabilitation centre<br><br>Mean age 61.3	- Part of a pilot study to find a suitable outcome measure for this sample<br>- One OT assessed all participants within one week of admission and discharge<br>- Calculated Standard error of measurement (SEM) based on Ottenbacher and colleagues [48]<br>- SEM = SD root (1-r), were SD and r were test retest reliability of review, SEM = 13 (used this value as minimum important difference)	- Results, mean difference of admission and discharge scores = 19.3<br>- Only 55% exceeded a difference of 13 points<br>- 26% of the sample had admission scores above 113, therefore no possibility of a >13 point difference – evidence of a ceiling effect in this population<br>- When the 11 individuals that scored above 113 on admission were removed from the sample, 74% had a difference >13 points<br>- Concluded: due to the ceiling effect, it is not suitable to use the FIM to measure change in this population
van der Putten et al., 1999	201 MS patients and 82 poststroke patients from an inpatient neurorehabilitation<br><br>Mean age 48	- Compared the appropriateness and responsiveness of the FIM and the BI<br>- Assessed all participants within 96 hours of admission and discharge with both tools<br>- Appropriateness was examined based on score ranges, means, SD, and floor and ceiling effects, where floor and ceiling effects exceeding 20% were considered significant<br>- Responsiveness was calculated using the ES	- FIM total, FIM motor and BI all had a wide range of scores (21-123, 13-91, 0-20), mean scores were near the midpoint of the scale (90.0, 57.6, 11.7) and small floor and ceiling effects (ranged from 0-8.5%)<br>- FIM cog scores had low variability and were highly concentrated around the upper range of the scale (ceiling effect 13.4-17.9%) especially in MS patients<br>- All ES were positive, indicating only improvement<br>- ES for the FIM total, FIM motor and BI were all similar and higher in stroke patients (0.82, 0.91, 0.95) than MS patients (0.30, 0.34, 0.37); concluded that these scales are responsive<br>- ES of the FIM cog was very low, concluded that this scale was not responsive
Wallace et al.,	372 stroke patients	- Assessed the responsiveness of the BI and FIM motor for evaluating recovery from	Measures of Responsiveness

<table>
<tr><td>2002</td><td>from a inpatient rehabilitation facility

Mean age 69.7</td><td>stroke over the 1-3 month post-stroke period
- Also assessed the impact of different methods for measuring responsiveness on instrument comparison
- Trained nurses/physical therapist assessed all patients with the Rankin Scale, BI and FIM motor at baseline 1 and 3 months post stroke
- Used the Rankin Scale to define clinically meaning full change
- Divided participants in 3 groups based on the results of the Rankin scale
- those who improved were labelled the "changers"
- those who did not change were labelled "unchangers"
- those who declined were excluded
- Of the 459 eligible participants,154 were changers, 218 were unchangers and 87 were excluded
- Calculated responsiveness by: area under ROC curve, Guyatt's effect size, paired t test, standard response mean, Kazis effect size, and mixed model adjusted t statistic</td><td><table>
<tr><th></th><th>FIM motor</th><th>BI</th></tr>
<tr><td>ROC curve</td><td>0.675</td><td>0.650</td></tr>
<tr><td>Guyatt effect size</td><td>1.29</td><td>1.29</td></tr>
<tr><td>Paired t test</td><td>12.0</td><td>12.1</td></tr>
<tr><td>SRM</td><td>0.62</td><td>0.63</td></tr>
<tr><td>Kazis effect size</td><td>0.31</td><td>0.28</td></tr>
<tr><td>Mixed model adjustable t-statistic</td><td>10.6</td><td>10.9</td></tr>
</table>
- Both instruments are able to demonstrate change, no measure is clearly superior
- Consistent findings with all methods of measuring responsiveness, no superior measures</td></tr>
</table>

Table S5: Summary of articles that investigated the reliability of the interRAI/MDS

<table>
<tr><th>Reference</th><th>Sample and Setting</th><th>Description</th><th>Results</th></tr>
<tr><td>Carpenter et al., 2001</td><td>233 patient receiving acute care

Mean age 78</td><td>- 2 independent assessments (MDS-AC), within 24 hours, by a trained nurse or doctor
-153 patients within 48hours of admission, 80 patients within 48hours of discharge
- Calculated percent agreement, κ (binary items) and wκ (wκ, multi-level items) to estimate interrater reliability (where κ >0.4 "sufficient for practical use")
- For average reliability estimates, items regarding pre-hospital status and inpatient status were separated</td><td>- Excluded items where 90% or more of the subjects had the same response
- Average reliability estimates
- Pre-hospital 0.57
- In-hospital 0.58
- Exact percent agreement was 83% for pre-hospital ratings and 79% for in-hospital ratings
- Concluded: the MDS AC achieved high reliability</td></tr>
<tr><td>Casten et al., 1998</td><td>733 residents from a nursing home with probable dementia

Mean age 84.50</td><td>- Study aimed to mimic the clinical environment (ie training, time, etc.)
- 2 independent assessments (MDS-2.0), both completed within 24hours
- 1st rater = Care Coordinator, 2nd rater = Nurse from the institution's quality assessment department
- Calculated PCC and κ to investigate interrater reliability</td><td>Interrater Reliability Estimates<table>
<tr><th></th><th>r</th><th>κ</th></tr>
<tr><td>Cognition</td><td>0.80</td><td>0.63</td></tr>
<tr><td>ADL (10 items)</td><td>0.99</td><td>0.61</td></tr>
<tr><td>Time use</td><td>0.75</td><td>0.75</td></tr>
<tr><td>Social quality</td><td>0.94</td><td>0.74</td></tr>
<tr><td>Depression</td><td>0.89</td><td>0.56</td></tr>
<tr><td>Problem behaviours</td><td>0.95</td><td>0.84</td></tr>
</table>
- Concluded: correlations between raters were high and kappas were "at least acceptable and generally high"</td></tr>
<tr><td>Graney & Engle, 2000</td><td>42 residents from 2 nursing homes

Mean age 67.8</td><td>- Studied the equivalence of 3 independent assessments of the 13 MDS 2.0 ADL items (interrater reliability)
- Each resident was assessed 3 times during a day shift for 7 days within 14 days of admission as per MDS directions
- Raters: trained interviewers
- Evaluated reliability using a two-way ANOVA for ranks where each participant was evaluated for evidence of within-subject difference</td><td>- There were no statistically significant within-subject difference among the 3 assessment for any of the 13 ADL measures (range of p values for the 13 items 0.305-0.996)
- Concluded: fewer than the required 21 assessments (3/day for 7 days), can be used for accurate evaluation of residents' ADL performance using the MDS 2.0</td></tr>
</table>

<table>
<tr>
<td>Gruber-Baldini et al., 2000</td>
<td>1900 residents from 59 nursing homes

Mean age 81.6</td>
<td>- All residents were assessed with the MDS-Cognitive Performance Scale (CPS) and MDS-Cognition Scale (MDS-COGS) (MDS 2.0) within 21-65 days of admission by a trained member of the nursing home staff
- Examined internal consistency using Cronbach’s alpha and PCC to measure item-total correlations</td>
<td><table>
<tr><td></td><td>Internal consistency</td><td>Item-total correlations</td></tr>
<tr><td>CPS</td><td>0.70</td><td>0.06 (comatose) – 0.67 (decision making)</td></tr>
<tr><td>MDS-COGS</td><td>0.85</td><td>0.32 (making oneself understood) – 0.81 (decision making skills)</td></tr>
</table>- The alpha of the CPS improved to 0.80 when the comatose item was removed</td>
</tr>
<tr>
<td>Hawes et al., 1995</td>
<td>123 residents from a nursing home</td>
<td>- Independently evaluated by 2 trained nurses (MDS 2.0)
- Calculated a Spearman Brown ICC for each item to estimate interrater reliability
- Defined excellent reliability as ICC >0.70 and adequate reliability as ICC>0.40</td>
<td>- Of all the items in the tool 89% ICC = 0.4 or higher, 63% ICC = 0.6 or higher
- Dropped 22 items due to poor reliability
- Of the 8 ADL items, all were found have excellent reliability, with an average reliability of 0.92
- Concluded: the reliability of MDS items are sufficient for research purposes</td>
</tr>
<tr>
<td>Hirdes et al., 2002</td>
<td>261 psychiatric patients in acute, long-term, geriatric, and forensic mental health beds in 14 hospitals

Mean age 45.7</td>
<td>- Two raters independently assessed each participant (MDS-MH) within 24 hours for acute patients and 7 days for long term, geriatric and forensic patients
- Raters: trained nurses, social workers and/or psychiatrists
- Calculated wκ (where >0.40 acceptable and >0.70 excellent) and percent agreement to estimate interrater reliability
- Select subscales (ADL-Long Form, IADL Summary, Depression Rating Scale) were evaluated for internal consistency using Cronbach’s alpha</td>
<td>- The average wκ for each section ranged from 0.39-0.78, with 1 (delirium), 23 and 5 sections having wκ values in the poor, adequate and excellent range respectively
- The percent agreement for each section ranged from 58-95.7, with 21 of the 29 sections having >80% agreement
Internal Consistency<table>
<tr><td></td><td>Number of items</td><td>Cronbach’s alpha</td></tr>
<tr><td>ADL-LF</td><td>7</td><td>0.95</td></tr>
<tr><td>IADL</td><td>6</td><td>0.92</td></tr>
<tr><td>DRS</td><td>7</td><td>0.77</td></tr>
</table>- Conclude: the majority of items demonstrated acceptable or higher average levels of interrater reliability</td>
</tr>
<tr>
<td>Hirdes et al., 2008</td>
<td>783 participants from 12 countries

246 LTCF, 220 HC, 126 PC, 102 PAC, 89 MH

Age: 9.9% <65, 57.5% 65-85, 32.6% >85</td>
<td>- Investigated the reliability of the items from 5 interRAI instruments supporting home care (MDS-HC), long term care (MDS-LTC), mental health (MDS-MH) palliative care (MDS-PC) and post-acute care (MDS-PAC)
- All participants were assessed with the appropriate instrument for their setting by 2 trained health professionals (ordinary clinical staff, external research staff, or both) independently within 72 hours
- Analysed interrater reliability using κ for binary items and wκ for multi-level items (where <0.40 poor, 0.41-0.60 moderate, 0.61-0.80 substantial, >0.81 almost perfect)</td>
<td>- For the 161 items common to two or more instruments, mean κ = 0.75, LTCF had the highest mean κ (0.74) and the HC instrument had the lowest (0.69)
- For specialized items (unique to individual instruments varied from 8-170 items) the PAC had the highest mean κ value (0.73) and the other instruments ranged between 0.63 and 0.68
- ADL items were amongst the most reliable values in the total sample with κ of 0.80 of better
- The lowest mean κ values for individual items had moderated to substantial agreement (κ 0.60-0.70)
- The large majority of items performed well in all 5 settings
- Concluded: interRAI instruments exceeded standard cut-offs for acceptable reliability and retained their reliability across care setting which provides evidence to support cross domain application of the instruments as part of an integrated health information system</td>
</tr>
<tr>
<td>Kwan et al., 2000</td>
<td>179 participants receiving home care

Mean age 72.9</td>
<td>- Investigated the internal consistency for summative outcome measures from the Chinese version of the interRAI HC
- Participants were assessed by two trained research assistants
- Calculated Cronbach Coefficient</td>
<td>- Of the outcome measures investigated, Cronbach Coefficient ranged from 0.49-0.80<table>
<tr><td>Outcome Measure</td><td>Cronbach Coefficient</td></tr>
<tr><td>IADL-capacity</td><td>0.68</td></tr>
<tr><td>IADL involvement</td><td>0.68</td></tr>
<tr><td>Stamina</td><td>0.49</td></tr>
<tr><td>Communication</td><td>0.80</td></tr>
<tr><td>Mood</td><td>0.69</td></tr>
<tr><td>Pain</td><td>0.73</td></tr>
</table>- Concluded: internal consistency was acceptable to consider the potential of adopting MDS-HC for Chinese population</td>
</tr>
<tr>
<td>Morris et al., 1990</td>
<td>383 residents from a nursing home</td>
<td>- Each resident was independently assessed (MDS-2.0) by two trained nurses
- 1 worked at that facility, 1 employed by the project
- 3 strategies to determine interrater reliability
1) percentage agreement
2)association b/w the judgements of pairs of assessors for the same items</td>
<td>- Overall >55% of items tested achieve reliabilities of 0.40 (ICC)
Reliability of ADL items<table>
<tr><td></td><td>Dichotomous ADL items</td><td>Multilevel ADL items</td></tr>
<tr><td>1) Percent Agreement</td><td>78.0-92.4</td><td>perfect:33.3-55.2, within 1: 73.3-89.6</td></tr>
<tr><td>2) Association</td><td>0.0-0.19</td><td>0.61-0.88</td></tr>
</table></td>
</tr>
</table>

<table>
<tr>
<td></td>
<td></td>
<td>- Phi associative statistic dichotomous item, and Rio statistic for multi-level items
3) ICC
- Items with low interrater reliability (<0.40) were eliminated unless they had strong clinical relevance</td>
<td><table><tr><td>3) ICC</td><td>-0.15-0.32</td><td>0.75-0.81</td></tr></table>- All dichotomous ADL items had low reliabilities
- bedfast was the only dichotomous item retained (and altered) as it is important for care planning, all others were dropped
- All multi-level ADL items were found to have high interrater reliability</td>
</tr>
<tr>
<td>Morris et al., 1997a</td>
<td>241 clients receiving home care
187 residents from a nursing home

Mean age 79.6</td>
<td>- Compared the interrater reliability of items in the MDS-2.0 and MDS-HC
- Independent assessments by 2 trained clinicians within a 7 day period
- Calculated wκ for each item</td>
<td>- For the items contained in both scales (47% of the items on the MDS-HC)
Mean wκ MDS 2.0 = 0.75 MDS HC = 0.74
- For the items contained in the MDS-HC but not in the MDS2.0
Mean wκ = 0.70
- Concluded: MDS items perform equally as well in a home care setting as in a nursing home</td>
</tr>
<tr>
<td>Morris et al., 1997b</td>
<td>187 residents from 21 nursing facilities

Mean age 80.6</td>
<td>- Each resident had independent dual assessments using a draft version MDS 2.0 administer by trained nurses
- Calculated wκ to estimate interrater reliability (where <0.40 poor, 0.40-0.75 adequate, >0.75 excellent)</td>
<td>- Of the 42 new items added 1, 20 and 21 had poor, adequate, and excellent reliability respectively
- The reliability of the revised items ranged from 0.33-0.72 and was significantly higher than the reliabilities for the items they replaced.
- For the 82 items that did not change, revisions to process instructions, item definitions, or examples resulted in an 18% increase in the average wκ from 0.67 to 0.79
- Concluded: the findings support the reliability of the new and revised assessment items</td>
</tr>
<tr>
<td>Morris et al., 1999</td>
<td>175,920 residents from a nursing home</td>
<td>- Independent assessments (MDS 2.0) by 2 trained nurses within a 7 day period
- Examined the internal consistency of the ADL Long Form and ADL Short Form with Cronbach's alpha
- Calculated wκ for each ADL item separately to investigate interrater reliability
- Defined wκ over 0.75 evidence of excellent reliability</td>
<td>- ADL Long Form α = 0.94, ADL Short Form α = 0.90

- wκ range for ADL items 0.87-0.94 (excellent reliability)</td>
</tr>
<tr>
<td>Phillips et al., 1993</td>
<td>147 residents from a nursing home</td>
<td>- Assessed the impact of the patients cognitive status on the interrater reliability of the MDS 2.0
- Selected a purposive sample of 40 MDS 2.0 items
- these items were used to construct 5 summary indices; all items, functional status
and continence, communication and sensory abilities, psychotropic drug and restraint use, and sad mood and behaviour
- Used the CPS to classify residents by cognitive status, 2 groups each intact and impaired
- Independent assessment by 2 trained nurses
- Calculated an indices of disagreement by adding the total number of disagreements
- For multilevel items, counted exact agreement and disagreement only, did not account for gradations of disagreement
- Used ANOVA to determine if there was a statistically significant difference b/w the number of disagreements in the intact and impaired groups
- Developed a series of binary multivariate models to estimate the impact of patient's cognitive status on the item reliability relative to other possible sources of error (type of assessor, resident's ADL needs and resident's LOS)</td>
<td>Average disagreement b/w assessors<table><tr><td></td><td>Cognitively intact</td><td>Cognitively impaired</td><td>Statistically significant</td></tr><tr><td>All items</td><td>7.4</td><td>10.3</td><td><.001</td></tr><tr><td>Functional Status</td><td>2.7</td><td>3.6</td><td>.02</td></tr><tr><td>Communication</td><td>0.9</td><td>2.1</td><td><.001</td></tr><tr><td>Drugs/restraints</td><td>0.4</td><td>0.6</td><td>.05</td></tr><tr><td>Mood/behaviour</td><td>3.5</td><td>3.8</td><td>.48 (not statistically significant)</td></tr></table>- There was a significant effect of cognitive function for four of the five indices
- the residents cognitive function is inversely related to interrater reliability
- for all items, in residents who were cognitively impaired the level of disagreement increased by 40%
- Items that required subjective assessment decisions were more affected than those that relied on medical records
- The multivariate models were completely consistent with the initial findings, in the same four indices cognitive impairment had a significant impact on reliability (accounted for the most of the variance)</td>
</tr>
<tr>
<td>Phillips & Morris, 1997</td>
<td>4 separate databases of nursing home residents
1) Research database, n = 2,000
2) Kansas database, n = 27,000
3) Mississippi database, n = 19,000
4) Washington</td>
<td>- Compared the internal consistency of MDS 2.0 data collected during a research study with that from 3 administrative data bases in which the data were collected by facility members
- Analyzed 7 cognitive functioning items and 7 ADL items
- Calculated PCC to investigate internal consistency of the items and scales</td>
<td>Internal consistency (α)<table><tr><td></td><td>Research</td><td></td><td>Kansas</td><td></td><td>Mississippi</td><td></td><td>Washington</td><td></td></tr><tr><td></td><td>COG</td><td>ADL</td><td>COG</td><td>ADL</td><td>COG</td><td>ADL</td><td>COG</td><td>ADL</td></tr><tr><td>Correlations among items</td><td>0.48-0.71</td><td>0.62-0.85</td><td>0.51-0.74</td><td>0.66-0.87</td><td>0.51-0.72</td><td>0.66-0.89</td><td>0.46-0.72</td><td>0.58-0.85</td></tr><tr><td>Alphas for additive scales</td><td>0.91</td><td>0.95</td><td>0.91</td><td>0.96</td><td>0.92</td><td>0.96</td><td>0.91</td><td>0.94</td></tr><tr><td>Item correlations with additive scales</td><td>0.63-0.81</td><td>0.63-0.81</td><td>0.66-0.80</td><td>0.71-0.90</td><td>0.67-0.85</td><td>0.74-0.90</td><td>0.71-0.84</td><td>0.77-0.90</td></tr></table>- Concluded: there is very little variation in the data provided from a research database and from</td>
</tr>
</table>

<table>
<tr><td></td><td>database, n = 6,000</td><td></td><td>clinical/administrative databases</td></tr>
<tr><td>Sgadari et al., 1997</td><td>Residents in nursing homes, accumulation of results from 7 countries

Age varied by country, range 24-129</td><td>- Each resident was independently assessed with the MDS 2.0 by 2 trained nurses within 1-14 days
- Calculated ICC to estimate interrater reliability</td><td>- Results ranged by country from 0.76 (Sweden) 0.97 (Denmark)
-Average reliability of ADL performance items range from 0.62-0.92 by country
- Concluded: “vast majority of RAI items achieve adequate to excellent reliability in all the countries”</td></tr>
<tr><td>Zimmerman et al., 2007</td><td>166 residents from 14 residential care/assisted living (RC/AL) facilities without a diagnosis of dementia

Mean age 83.6</td><td>- Investigated the inter and intrarater reliability of the MDS-COGS in screening for undetected dementia (MDS 2.0)
- Each resident was assessed twice by the 2 staff members involved in their care (test period 2-5 days)
- The two raters were not restricted from discussing the residents status but completed the MDS-COGS form independently
- 2 MDS-COGS cut points were assessed 0 (no impairment) vs 1 or more (any impairment) and 0 to 1 vs 2 or more
- Calculated unweighted κ to determine reliability</td><td><table>
<tr><td></td><td colspan="2">Interrater Reliability</td><td colspan="2">Intrarater Reliability</td></tr>
<tr><td>Cut point</td><td>κ</td><td>95% CI</td><td>κ</td><td>95% CI</td></tr>
<tr><td>0 vs >1</td><td>0.29</td><td>0.13-0.44</td><td>0.59</td><td>0.37-0.81</td></tr>
<tr><td>0-1 vs >2</td><td>0.46</td><td>0.30-0.63</td><td>0.43</td><td>0.43-0.76</td></tr>
</table>
- Concluded: in this population of raters the MDS-COGS had moderate reliability</td></tr>
</table>

Table S6: Summary of articles that investigated the validity of the interRAI/MDS

<table>
<tr><th>Reference</th><th>Sample and Setting</th><th>Description</th><th>Results</th></tr>
<tr><td>Carpenter et al., 2006</td><td>7001 moderately demented and 4616 severely demented residents of a nursing home

Mean age 85.6</td><td>- Aimed to assess the responsiveness of the MDS-ADL Long Form in adults with moderate and severe dementia
- Used CPS to determine severity of dementia; moderate CPS score of 3,severe CPS score of 4 or 5
- Excluded any residents with known comorbid conditions
- Defined clinically meaningful change as a one point change on the MDS-ADL, based on nurse debriefing sessions during the tools development [25]
- Calculated mean change from baseline to 3 and 6 months, separately for moderately and severe dementia</td><td>- The moderately impaired group showed the greatest change in early and middle level ADL items, while the severe group showed the greatest loss in late loss ADL items
- For the moderately impaired group the average ADL decline at 3 months was 1.02 points and at 6 months 1.78 (SD 4.4) points, (95% CI 1.67-1.91)
- For the severely impaired group the average ADL decline at 3 months was 1.07 points and at 6 months 1.70 (SD 3.9)points, (95% CI 1.59-1.83)
- Concluded: the instrument was capable of detecting clinically meaningful change in physically function in nursing home residents with moderate to severe dementia</td></tr>
<tr><td>Casten et al., 1998</td><td>733 residents of a nursing home

Mean age 84.50</td><td>- Confirmatory factor analysis (MDS 2.0)
- Hypothesized factor model has 6 factors; cognition, activities of daily living, time use, social quality, depression and problem behaviours
- Separated sample into 4 groups
- Used Group 1 and 2 to test the reproducibility of the hypothesized factor model in two heterogeneous groups
- randomized the groups by level of cognitive impairment
- hypothesize a factor model and develop it using a maximum likelihood solution adjusted for sample 1
- used sample 2 to test the model
- Group 3 and 4 were divided by cognitive status, intact (higher cognitive functioning, S3) and impaired (lower cognitive functioning, S4), to compare the factor loading patterns related to cognitive status</td><td>- 5 of the 6 factors were confirmed in the high functioning residents and residents randomized by cognitive status
- social quality was the 1 factor not confirmed in these groups
- 0 of the 6 factors were confirmed in the low functioning group
- Specifically for the ADL factor, when the intact group was compared to the impaired group they had “radically different” structure (chi-square = 76.6, p<.001)
- Concluded: error is introduced when the MDS is used to compare groups with different cognitive status</td></tr>
<tr><td>Cohen-Mansfield et al., 1999</td><td>290 residents of a nursing home

Mean age 87</td><td>- Investigated the correlation between the MDS 2.0 CPS and MDS-COGS with the Mini-Mental State Exam (MMSE) and the Global Deterioration Scale (GDS)
- All participants were assessed with all 4 instruments on admission by trained nurses
- Calculated PCC to investigate relationship</td><td><table>
<tr><td></td><td>CPS</td><td>MDS-COGS</td></tr>
<tr><td>MMSE</td><td>-0.71</td><td>-0.75</td></tr>
</table></td></tr>
</table>

<table>
<tr><td></td><td></td><td></td><td><table><tr><td>GDS</td><td>0.75</td><td>0.77</td></tr></table>- The CPS and the MDS-COG correlate strongly (0.93)
- Both the CPS and the MDS-COGS are strongly correlated with the MMSE and GDS
- The MDS-COGS correlated to the MMSE and the GDS slightly higher than the CPS</td></tr>
<tr><td>Gruber-Baldini et al., 2000</td><td>1900 residents from 59 nursing homes

Mean age 81.6</td><td>- All residents were assessed with the CPS and MDS-COGS (MDS 2.0) within 21-65 days of admission by a trained nurse
- Additional data were collected from: interviews with a proxy family member, friend or other person who knew the resident prior to admission (Blessed Dementia Scale Changes in Everyday Activities and Difficulty Subscales, BC), a member of the nursing staff most familiar with the resident (Psychogeriatric Dependency Rating Scale, PGDRS and Katz ADL Scale) and the resident (Mini Mental State Exam, MMSE)
- Examined validity using PCC and f tests of means</td><td>- The correlation between the CPS and the MDS-COGS was 0.92
- The MDS-COGS and the CPS were correlated with the MMSE and the PGDRS orientation, ranging in absolute value from 0.63 to 0.68
- Assessing divergent validity, correlations of the MDS cognitive scales with the PGDRS behaviour ranged from 0.28-0.31 with more functional scales ranging from 0.37-0.50
- Concluded: compared with other instruments, the MDS-COGS and the CPS had moderate and similar validity for assessing cognitive impairment</td></tr>
<tr><td>Hartmaier et al., 1994</td><td>200 residents from 8 nursing homes

Mean age 80.5</td><td>- Aimed to develop a new, continuous scale to assess cognitive impairment using MDS items
- Each resident was assessed once with the Global Deterioration Scale (GDS) and the Mini Mental State Exam (MMSE) by a medical student
- Independently, a geriatric nurse assessed each resident on the CPS and additional MDS items thought to be related to cognitive functioning
- Prior to the analysis, the sample was randomly split into two groups of 133 and 67 to allow for instrument development with the first group and validation with the second group
- Investigated the agreement between the scales using weighted and unweighted κ
- Performed a logistic regression analysis to identify additional (to the CPS) MDS items predictive of GDS stages of cognitive impairment
- Modified the CPS with additional MDS items until wκ with 4-stage GDS was maximized
- Examined the validity of the MDS-COGS (newly developed scale) against the GDS and MMSE in the second group by calculating Spearman correlation, weighted and unweighted κ, percent agreement, and sensitivity and specificity</td><td>- The GDS tended to classify subjects as more cognitively impaired than the CPS
- Fair agreement between GDS and CPS, wκ = 0.41 and percent agreement ranged from 0-50%
- In this population, further examination of the GDS revealed, mild to moderated cognitive impairment were not discriminated, instead the first 4 GDS stages were being lumped into one stage. Concluded that in this sample the GDS was not appropriately a 7-stage scale and continued the analysis using a 4-stage GDS scale.
- Found "substantial" agreement between the 4-stage GDS and the CPS (κ = 0.76), but percent agreement remained low (50% of less)
- Logistic regression revealed that many additional MDS items were predictive of GDS stages
- Yielded a maximum wκ with the GDS by including 8 MDS items assessed on a 10-point continuous scale, wκ = 0.82<table><tr><th></th><th>r</th><th>κ</th><th>wκ</th><th>sensitivity</th><th>specificity</th></tr><tr><td>GDS</td><td>0.92</td><td>0.68</td><td>0.80</td><td></td><td></td></tr><tr><td>MMSE</td><td>0.88</td><td>0.82</td><td></td><td>0.95</td><td>0.88</td></tr></table>Concluded: the MDS-COGS is a valid measure for the presence and severity of cognitive impairment in nursing home residents</td></tr>
<tr><td>Hartmaier et al., 1995</td><td>200 residents from 8 nursing homes

Mean age 80.5</td><td>- Each resident was assessed once on the Mini-Mental State Exam (MMSE) by a medical student
- Independent of the MMSE assessment, a geriatric research nurse assessed each resident on previously selected MDS cognitive items (CPS and addition MDS items considered to be related to cognitive impairment)
- Examined the correlation b/w the two instruments with the Spearman Correlation Coefficient
- Residents were classified into two groups 1) cognitively intact or 2) cognitively impaired based on crude (MMSE = 23) and education adjusted MMSE cut points
- The CPS cut point for cognitive impairment was 2 or more
- Assessed sensitivity and specificity based on MMSE and CPS cut points and developed ROC curves to illustrate the relationship
- Examined the level of agreement b/w the two instruments with κ coefficients of concordance and calculated positive predictive values (PPV) and negative predictive values (NPV)</td><td>- The average MMSE scores appeared to drop in a stepwise fashion across the seven CPS levels
- CPS level 0 (intact) and level 6 (very severe impairment) had an mean crude MMSE score of 24.2 (SD = 3.45), and 1.64 (SD = 3.53) respectively
- Spearman Correlation Coefficient, r = -0.863, p>0.001
- For crude MMSE and CPS scores sensitivity and specificity measures were above 0.80, and after adjusting for education level sensitivity and specificity measures for the CPS compared with the MMSE were both 0.94
- Reproducibility was κ = 0.85 (95% CI 0.72-0.98) and κ = 0.76 (95% CI 0.53-0.99) for high and low education respectively. After adjusting for education level, agreement between the CPS and the MMSE was κ = 0.82 (95% CI 0.68-0.96)
- The area under the ROC curve was 0.96 (95% CI 0.88-1.0), including excellent diagnostic accuracy of the CPS for the identification of cognitive impaired subjects
- PPV was 0.97 (95% CI 0.93-1.0) and the NPV was 0.80 (95% CI 0.69-0.91)
- Concluded: the CPS can be used to detect cognitive impairment of nursing home residents as defined by the MMSE</td></tr>
<tr><td>Hirdes et al., 2002</td><td>261 psychiatric patients in acute, long-term, geriatric, and forensic mental health beds in 14</td><td>- Aims to presents illustrative evidence for validity of the MDS-MH
- Two raters independently assessed each participant within 24 hours for acute patients and 7 days for long term geriatric and forensic patients
- Raters: trained nurses, social workers and/or psychiatrists
- Based on post hoc patterns of association investigated with ANOVA</td><td>- Patients age 65+ were significantly more cognitively impaired (higher CPS scores, t = 8.4, p<0.0001) and more disabled (higher ADL scores, t = 31.9 (p<0.0001) than their younger and middle-aged counterparts
- Participants that had suicide attempts in the past 12 months and those who has suicidal ideation in the last 30 days had higher depression than those not showing these indicators of suicidality</td></tr>
</table>

	hospitals<br><br>Mean age 45.7		(t=6.59, p<0.0001 and t=7.54,p<0.001, respectively)<br>- Clear tendency (χ^2 = 5.81, df = 1, p = 0.016) for those with multiple admissions to adhere to their medication regimens less than 80% of the time (revolving door syndrome)<br>- High score on CPS related to higher prevalence of behavioural disturbances<br>- Conclude: the above points are evidence of validity
Kwan et al., 2000	37 clients receiving home care<br><br>Age >65	- Investigated the concurrent validity of the Chinese version of the MDS-HC Clinical Assessment Protocols (CAPs) by comparing CAPs triggered by the MDS HC and CAPs diagnosed by a clinician<br>- Participants were assessed by two trained research assistants and a clinician blinded to the MDS assessment<br>- Agreement was examined by κ coefficient	- Of the 19 CAPs assessed, agreement was "perfect of substantial" for 4 CAPs (κ = 1.0-0.65), "slight" for 10 CAPs (κ = 0.54-0.27) and "poor" for 5 CAPs (κ = 0.19-0.00)<br>- Specifically for the ADL-rehabilitation potential CAP κ = 0.65 and for the Cognition CAP κ = 0.34<br>- Concluded: this level of agreement indicated a good potential of adopting the MDS-HC in the Chinese population
Landi et al., 2000	95 patients receiving home care<br><br>Mean age 77.4	- Assessed agreement of the MDS-ADL Long Form with the BI, MDS-IADL with the Lawton index and the CPS with the MMSE<br>- Every participant was independently assessed with all 4 instruments by trained nurses<br>- All assessment were completed within one week<br>- Analysed scatter plots and calculated the PCC for each pair	- All 3 scatter plots showed a linear relationship<br>- 0.74 MDS-ADL and BI (p<0.001)<br>- 0.81 MDS-IADL and Lawton index (p>0.001)<br>- 0.81 CPS and MMSE (p>0.001)<br>Concluded: there is a high association for all 3 comparisons
Lawton et al., 1998	513 nursing home residents<br><br>Divided into 2 groups, intact (n=260) and cognitively impaired (n=253)<br><br>Mean age 87	- Separate data sets of intact and impaired residents<br>Intact – able to give a self report<br>Impaired – not able to give a self report<br>- All subjects were assessed with 10 MDS-ADL items, the Lawton physical self-maintenance scale and a number of other MDS and non-MDS items/instruments not related to functional assessment (not described here)<br>- Separate analysis for intact and impaired group<br>- Hypothesized that the 2 instruments would correlate, calculated PCC	Intact group<br>r = 0.58<br><br>Impaired group<br>r = 0.79<br><br>Concluded: Moderate to high association b/w the scales provides evidence for validity
Morris et al., 1990	383 nursing home residents	- During the development of the MDS 2.0, nurses were asked to fill out a control form and a problem sheet to collect information regarding the instruments validity after each assessment<br>- the nurses commented on the relevance of each item (face validity)	- nurse's felt multicategory items were crucial to care planning<br>- they reported that a difference of one point defined an increase care requirement
Morris et al., 1994	Combined samples of 2, 172 residents from 269 nursing homes and 6, 663 residents from 176 nursing homes<br><br>Mean age 85	- Aimed to use MDS items to develop a valid hierarchical scale that described cognitive performance<br>- All residents were assessed with the MDS, MMSE and those who scored less than 10 on the MMSE were also assessed with the Test for Severe Impairment (TSI) by trained nursing staff<br>- A team of facility nurses also independently judged each resident's orientation status as an additional marker to assess the sensitivity and specificity of the MDS<br>- Automatic Interactions Detection (AID)(a type of cluster analysis) was used to develop classes of residents with distinct profiles as defined by the cognitive criterion measures of the MMSE, TSI and a combination of the two instruments (dependent variables)<br>- Theoretically defined cognitive and ADL measures on the MDS were used as independent variables for the model	- The accepted model (CPS) required 5 MDS variables (short term memory, cognitive skills for decision making, coma, making self understood and eating) with 7 response options that move progressively from relative independence (level 0) to extreme cognitive impairment (level 6)<br>- Each response option is statistically distinct based on the AID<br>- Based on judgements of facility nurses, 42% of the derivation sample were oriented, 30% were partially disoriented and 28% were disoriented<br>- The sensitivity and specificity of the CPS ranged from 0.82-0.95 and 0.88-0.92 respectively, relative to clinical judgement<br>- Concluded: the CPS provides a functional view of cognitive performance using readily available MDS data
Morris et al., 1999	175,920 residents from multiple nursing homes	- Tallied the distribution of response options for each item<br>- Aimed to give initial information on how the ADL items may be arranged hierarchically in relation to loss of function using exploratory factor analysis and hypothesis testing<br>- Examined what ADL items tended to moved from the independent to a non independent status first (estimate difficulty), and which residents were last able to retain an independent status	- Found all response options were used for every item<br>- 3 factors emerged in the factor analysis<br>Early loss: dressing and personal hygiene<br>Middle Loss: toilet use, transfer and locomotion<br>Late loss: bed mobility and eating<br>- "Middle Loss" category was separated into 2 clinically relevant categories; toilet use and movement

<table>
<tr>
<td></td>
<td></td>
<td>- Hypothesis that can separate items based on hierarchy of loss – starting with early loss ADLS items and continuing with middle and late loss items
- Also examining the probability of losing a specific ADL when you have already lost other ADLs (eg. if the participant has lost 2 other ADLs what is the likelihood that they have also lost hygiene)
- Compared data on 3 different MDS-ADL subscales scale (long form, short form, self performance hierarchy) to compare they ability to measure change over 3 and 12 month periods
- Defined clinically relevant change as a 4% decline of one standard deviation unit in 3 months and a 13% decline of one standard deviation unit in 12 months
- also compared the proportion of residents that showed any change on each subscale</td>
<td>
<table>
<tr><th></th><th>Mean Change</th><th>Percent Change in Standard Deviation Units</th></tr>
<tr><td>3-month change</td><td></td><td></td></tr>
<tr><td>ADL-Long Form</td><td>0.41</td><td>4.4</td></tr>
<tr><td>ADL Short Form</td><td>0.23</td><td>4.3</td></tr>
<tr><td>ADL Hierarchy</td><td>0.07</td><td>3.9</td></tr>
<tr><td>12-month change</td><td></td><td></td></tr>
<tr><td>ADL-Long Form</td><td>1.28</td><td>13.8</td></tr>
<tr><td>ADL Short Form</td><td>0.72</td><td>13.4</td></tr>
<tr><td>ADL Hierarchy</td><td>0.23</td><td>12.6</td></tr>
</table>
Concluded: all 3 scales are responsive, however the ADL long form is better at detecting minor, incremental changes</td>
</tr>
<tr>
<td>Morris et al., 2004</td>
<td>160 patients receiving home care (HC) and 350 patients from inpatient rehabilitation facilities, skilled nursing homes and long term care homes (SNH)

Mean age HC 78
SNH 80</td>
<td>- Explored the validity of summary scales created from MDS-HC and MDS-PAC items by investigating their association with established research and clinical assessment tools including: FIM, Outcome and Assessment Information Set (OASIS), Frailty and Injuries: Cooperative Studies of Intervention Techniques trials (FICSIT), Centre for Epidemiologic Studies-Depression Scale (CESD), Cornell Scale for Depression in Dementia, Mini-Mental State Exam (MMSE), Rehabilitation Institute of Chicago Functional Assessment Scale (RIC-FAS), Hearing Handicap Inventory Screening Version (HHIE-S), pain severity analog scale, supplementary interview on bowel and bladder function developed for this project and the Medical Outcomes Study Short form (SF-36)
- To minimize response burden the data collection instruments were shortened to a subset of the total scales and each respondent was assessed with 2-6 different subsets
- Used PCC to examine "correspondence between scale scores or individual items"
- Completed exploratory factor analyses and correlation matrixes when more than 2 summary measures were available</td>
<td>- The FIM and OASIS had similar levels of agreement with the MDS with most in the good to high range
- CPS was strongly correlated with the MMSE (0.69), OASIS cognitive function (0.77) and OASIS confusion (0.77)
- Forced 2-factor exploratory analysis of the MDS-ADL items resulted in the first factor being measured most strongly by the MDS-ADL-Hier. (r=0.96)
- the FIM-Self-Care and OASIS ADL summaries were highly correlated with this factor (r=0.58 and 0.61, respectively)
- Conclude: the results demonstrate the validity of MDS-derived summary measures with other research and standardized clinical assessment instruments</td>
</tr>
<tr>
<td>Phillips & Morris, 1997</td>
<td>4 separate databases of nursing home residents
1) Research database, n = 2,000
2) Kansas database, n = 27,000
3) Mississippi database, n = 19,000
4) Washington database, n = 6,000</td>
<td>- Compared validity of MDS 2.0 data collected during a research study with that from 3 administrative data bases collected by facility members
- Assessed 7 cognitive functioning items and 7 ADL items
- Calculated Spearman's Rho to investigate the correlation between the cognitive scale and the ADL scales
- Compared the internal consistency (α) of the ADL self-performance scale among populations with differing levels of cognitive impairment, types of raters and setting to investigate if ADL data are consistent across the subpopulations</td>
<td>- The correlation of the cognitive scale with the ADL scale was 0.50, 0.47, 0.55 and 0.44 in the Research, Kansas, Mississippi and Washington data respectively
<table>
<tr><th></th><th>Research data</th><th>Kansas data</th><th>Mississippi data</th><th>Washington data</th></tr>
<tr><td>Relatively impaired</td><td>0.92</td><td>0.94</td><td>0.94</td><td>0.92</td></tr>
<tr><td>Moderately impaired</td><td>0.92</td><td>0.94</td><td>0.94</td><td>0.93</td></tr>
<tr><td>Severely impaired</td><td>0.92</td><td>0.94</td><td>0.93</td><td>0.93</td></tr>
</table>
- Cognitive scale is moderately correlated with the ADL scale
- The MDS 2.0 provides consistent ADL data across 3 impairment subgroups
- Alpha coefficients do not vary across the 4 databases
- Concluded that there is very little variation in the data provided from a research database and from clinical/administrative databases</td>
</tr>
<tr>
<td>Snowden et al., 1999</td>
<td>140 nursing home residents enrolled in the Alzheimer's Disease Patient Registry

Mean age 83.4</td>
<td>- Aimed to investigate the association b/w MDS 2.0 subscales to comparable subscales from Alzheimer's Disease Patient Registry (ADPR) Measurement
- CPS vs MMSE
- MDS behaviours domain score (BDS) vs ADPR Physician behaviour checklist(PBC)
- MDS-ADL vs Dementia Rating Scale (DRS for ADLs)
- Maximum 90 days between assessments (mean 20.9 day, SD 22.9 days)
- Assessment completed by a research nurse via interviews with the patient's family and nursing staff
- 60 of the 140 residents were assessed at baseline and follow-up (average followuo</td>
<td>Association (ICC)
- CPS vs MMSE = 0.45
- MDS-BDS vs ADPR Physician Behaviour checklist (PBC) scores = 0.50
- MDS-ADL vs Dementia Rating Scale (DRS) for ADLs = 0.59
- Repeated calculations using only APDR items that appeared to measure the same construct as the MDS items, only slight improvements on association

Responsiveness
- CPS (ES = 0.60) > MMSE (ES = 0.39)
- MDS-ADL (ES 0.024) < DRS-ADL (0.77)</td>
</tr>
</table>

<table>
<tr><td></td><td></td><td>636 days (SD 131 days)
- Calculated Spearman's Correlation Coefficients to estimate correlation (where >0.80 excellent, 0.6-0.79 good, 0.4-0.59 fair, <0.40 poor) and ES to investigate responsiveness</td><td>- MDS-BDS (ES 0.058) < ADPR Physician Behaviour Checklist (0.065)
- the ES of the DRS was more than 10 times greater than the MDS-ADL, a sample size of >3000 would be required to measure change using the MDS-ADL</td></tr>
<tr><td>van der Steen et al., 2006</td><td>175 residents from a nursing home with moderate to severe dementia

Mean age: 62.7</td><td>- Used the Bedford Alzheimer Nursing Severity-Scale (BAN-S) as a standard for defining severe dementia, against MDS-based definitions
- Aimed to propose a new definition of severe dementia, based on MDS data
- Participants were assessed with the MDS, BANS-S and MMSE within a four-week period by nursing home staff (BANS-S and MDS)
- Used independent samples t-tests, Pearson's chi-square and κ to investigate the association b/w the measures</td><td>- CPS scores were driven by only 3 of the 5 component items since 0 residents were comatose and only 3 lacked shot term memory
- PCC b/w CPS and BANS-S scores was +0.50
- Half of all residents were assigned CPS scores of 5
- Mean BANS-S score increased with CPS score
- Within the CPS categories, BANS-S score varied widely
- CPS scored many more residents as severely cognitively impaired than the BANS-S
- poor correlation b/w the CPS and the BANS-S score when CPS was over 5 (κ 0.36)
- Addition of an ADL component to the CPS definition allows for improved distinction b/w moderate and severe dementia
- Proposed a CPS score of 5 or 6 with a minimum score of at least 10 points on the MDS ADL-short form as an MDS-bas3ed definition of severe dementia</td></tr>
<tr><td>Zimmerman et al., 2007</td><td>166 residents from 14 residential care/assisted living (RC/AL) facilities without a diagnosis of dementia

Mean age 83.6</td><td>- Investigated the sensitivity and specificity of the MDS-COGS in screening for undetected dementia
- Each resident was first assessed with the MDS-COGS by the staff member who was most involved in their care and then underwent a neurological assessment by a trained psychologist
- 2 MDS-COGS cut points were assessed 0 (no impairment) vs 1 or more (any impairment) and 0 to 1 vs 2 or more
- Calculated positive and negative agreement to estimate sensitivity and specificity</td><td><table><tr><td>Cut point</td><td>Sensitivity</td><td>95% CI</td><td>Specificity</td><td>95% CI</td></tr><tr><td>0 vs >1</td><td>0.67</td><td>0.55-0.80</td><td>0.84</td><td>0.76-0.91</td></tr><tr><td>0-1 vs >2</td><td>0.49</td><td>0.36-0.62</td><td>0.97</td><td>0.93-1.00</td></tr></table>- The neurologist determined that 55 participants had probable dementia
- 19% of those with an MDS-COGS score of 0 had a probable diagnosis of dementia, increasing to 46%, 78%, 91% and 100% as the MDS-COGS scores increased 1, 2, 3, and 4 or more
- The first cut point provides the highest sensitivity but is less specific and the second cut point provides the highest specificity but is less sensitive
- Concluded: the MDS-COGS will identify with high specificity a subset of residents with undetected dementia but caution needs to be exercised due to its low sensitivity as some with milder dementia will not be detected</td></tr>
<tr><td>Jette et al., 2003

*</td><td>485 patients receiving post acute care

199 from acute inpatient rehabilitation, 90 from transitional care units, 90 from community ambulatory services and 106 from home care

Mean age 62.7</td><td>- Investigate whether the setting specific functional assessment instruments used in post acute care (PAC) each have fundamental differences that prevent their applicability across diagnosis, over time and across different PAC settings
- Compared the FIM, OASIS, MDS 2.0 and PF-10
- Stratified patients by impairment group; neurological, musculoskeletal and medically complex and by severity of impairment; slight, moderate and severe to ensure a representative sample
- Collected standardized assessment information via retrospective chart review when available; FIM total for patients in inpatient rehabilitation, 19 MDS items for persons in skilled nursing facilities and 19 OASIS-ADL items for persons receiving home care
- Administer 10 physical functioning SF-36 to individuals receiving outpatient services where no standardized assessment information was available
- Assessed all patients via personal interview with a newly developed core set of 58 activity items
- Used minimum and maximum threshold values for each instrument to determine range of content coverage
- Analysed item characteristic curves to determine the degree and location of information provided by each scale</td><td>- Across all instruments cognitive, communication, bowel and bladder were the easiest items for this sample to perform (require less functional ability to perform)
- PF-10 contained the most difficult items (require more functional ability to perform)
- "a substantial number" of FIM, OASIS and MDS items required an average range of functional ability – clustered around the midpoint of the functional ability continuum
- Across these four instruments there was "substantial overlap" in content
- The range of coverage was greatest for the MDS and the OASIS
- Measurement precision
- OASIS and MDS, greatest at the low end of the functional ability dimension
- FIM, greatest at the low to moderate point on functional continuum
- PF, greatest at the high end of the functional continuum
- Specifically, the FIM was found to be most precise and relevant for PAC inpatients(low end of functional continuum) and the MDS covered content from the mid portion of the continuum having less coverage at both the high and low end
- Concluded: each of the four instruments are well suited for its primary application, none appear to be well equipped across all settings</td></tr>
</table>

* Jette et al., 2003 contains validity information for both the FIM and the MDS
